# Canadian Potential Healthcare and Societal Cost Savings from Consumption of Pulses: A Cost-Of-Illness Analysis

**DOI:** 10.3390/nu9070793

**Published:** 2017-07-22

**Authors:** Mohammad M. H. Abdullah, Christopher P. F. Marinangeli, Peter J. H. Jones, Jared G. Carlberg

**Affiliations:** 1 Department of Human Nutritional Sciences and Richardson Centre for Functional Foods and Nutraceuticals, University of Manitoba, Winnipeg, MB R3T 6C5, Canada; umabdul7@myumanitoba.ca (M.M.H.A.); Peter.Jones@umanitoba.ca (P.J.H.J.); 2 Department of Food Science and Nutrition, Kuwait University, Kuwait City 10002, Kuwait; 3 Pulse Canada, Winnipeg, MB R2C 0A1, Canada; cmarinangeli@pulsecanada.com; 4 Department of Agribusiness & Agricultural Economics, University of Manitoba, Winnipeg, MB R2C 0A1, Canada

**Keywords:** dietary pulses, diabetes, cardiovascular disease, healthcare cost savings, nutrition economics

## Abstract

Consumption of dietary pulses, including beans, peas and lentils, is recommended by health authorities across jurisdictions for their nutritional value and effectiveness in helping to prevent and manage major diet-related illnesses of significant socioeconomic burden. The aim of this study was to estimate the potential annual healthcare and societal cost savings relevant to rates of reduction in complications from type 2 diabetes (T2D) and incidence of cardiovascular disease (CVD) following a low glycemic index (GI) or high fiber diet that includes pulses, or 100 g/day pulse intake in Canada, respectively. A four-step cost-of-illness analysis was conducted to: (1) estimate the proportions of individuals who are likely to consume pulses; (2) evaluate the reductions in established risk factors for T2D and CVD; (3) assess the percent reduction in incidences or complications of the diseases of interest; and (4) calculate the potential annual savings in relevant healthcare and related costs. A low GI or high fiber diet that includes pulses and 100 g/day pulse intake were shown to potentially yield Can$6.2 (95% CI $2.6–$9.9) to Can$62.4 (95% CI $26–$98.8) and Can$31.6 (95% CI $11.1–$52) to Can$315.5 (95% CI $110.6–$520.4) million in savings on annual healthcare and related costs of T2D and CVD, respectively. Specific provincial/territorial analyses suggested annual T2D and CVD related cost savings that ranged from up to Can$0.2 million in some provinces to up to Can$135 million in others. In conclusion, with regular consumption of pulse crops, there is a potential opportunity to facilitate T2D and CVD related socioeconomic cost savings that could be applied to Canadian healthcare or re-assigned to other priority domains. Whether these potential cost savings will be offset by other healthcare costs associated with longevity and diseases of the elderly is to be investigated over the long term.

## 1. Introduction

Dietary pulses, including several varieties of dry beans, peas, chickpeas, and lentils, are the edible seeds of members of the legume family [1]. Pulse crops have a long tradition of being part of human diets, with historical records dating as far back as 10,000 years before present [2], and are rich sources of dietary fiber, protein, antioxidants, minerals (i.e., iron, zinc, magnesium and phosphorous), as well as folate and other B-vitamins [3]. For this reason and for their actions in reducing risk or managing major diet-related chronic disorders, such as obesity, diabetes mellitus (DM), and cardiovascular disease (CVD) [4,5,6,7], consumption of dietary pulses is recommended by health authorities worldwide [8,9,10,11,12,13,14,15,16].

Canada’s Food Guide [4] encourages regular consumption of cooked pulses as part of the recommended 2–3 daily servings of foods from the “Meat and Alternatives” group for the adult population. Within Canada’s Food Guide, ¾ cup (175 mL) represents one serving of cooked pulses. Although Canada is a world leader in the production and sale of pulses [17], and pulses are plentiful within the Canadian marketplace, their consumption remains low, with only 13% of the adult population reporting consumption of pulses (at an average of 113 g) on any given day [18].

The economic burden of diet-related disorders is recognized worldwide. For instance, diabetes-related expenditures reached over US$600 billion in 2014 and have been forecast to increase by 40%, from US$500 billion to over US$700 billion, between 2010 and 2030 [19]. Similarly, it has been estimated that the global expenditures related to CVD will reach US$20 trillion between 2010 and 2030 [19]. Healthy dietary behaviors have been demonstrated to contribute to substantial savings on healthcare and related costs [20,21,22,23,24]. In this regard, epidemiological, randomized controlled, and meta-analytic research support the beneficial effect of dietary pulse consumption on established risk factors for type 2 diabetes (T2D) [25,26,27,28,29,30] and CVD [31,32,33,34,35]. As such, increasing consumption of pulses has the potential to be a powerful tool for policymakers to help manage healthcare resources associated with these illnesses. Data on the economic value of greater habitual or recommended pulse consumption are, however, lacking.

Given that dietary pulses are a readily available and affordable source of nutrition within the Canadian marketplace, the objective of this research was to assess the potential annual savings in healthcare and productivity costs that would arise from reduced incidence of CVD and management of T2D following increased pulse consumption, or adoption of low glycemic index (GI) or high fiber diets that include pulses in Canada, respectively.

## 2. Materials and Methods

### 2.1. Financial Model Design

A cost-of-illness analysis [36,37] involving four steps was used for this research; a detailed description of each step is provided below. Step 1 estimated the *dietary intake success rate (%)*; the proportion of individuals likely to consume a reasonable daily serving of pulses in Canada. Step 2 estimated the *disease biomarker reduction rate (absolute unit or percent).* Glycated hemoglobin (HbA1c) was used as a biomarker of blood glucose management and risk factor for complications associated with T2D. LDL-cholesterol (LDL-C) concentrations and systolic blood pressure (SBP) were used as biomarkers of CVD risk. Risk factor reduction per serving of pulses was subsequently calculated for CVD incidence reduction, while reduced risk of complications associated with lower HbA1c from the adoption of a low GI or high fiber diet was assessed for DM patients. Step 3 estimated the *disease incidence and complication reduction (%)*; the percent reduction in T2D-related complications from low GI or high fiber diets and CVD incidence risk rates with daily consumption of pulses. Finally, Step 4 calculated the *disease cost savings (Can$)*; the potential annual healthcare and related cost savings associated with the estimated reduction in complications from T2D and incidence of CVD. Scientific and monetary data that formed the basis of estimates in the model were derived upon review of the present literature and recent healthcare cost figures from national databases.

For savings in T2D-related healthcare costs, the effects of pulses on the management of HbA1c concentrations were estimated under the context of using pulses as part of low GI or high fiber diets. Conversely, CVD risk factor reduction was based on a daily serving of pulses, and, for the purpose of this study, a daily level of pulses that could be reasonably consumed by Canadians was employed. Canada’s Food Guide indicates that ¾ of a cup, or ~150 g (Pulse Canada unpublished data), is a serving of pulses [8]. However, given the wide range of daily intake levels for whole pulses [18] and their relatively low consumption frequency in Canada [38], 150 g/day pulses is likely a target consumption level that would be difficult for most Canadians. Cross-sectional data from the 2004 Canadian Community Health Survey suggested 113 g as the average amount of pulses consumed by adults on any given day [18]. In the same study, 66–137 g and 99 g pulses were the range and mean consumption level for the third quartile, respectively [18]. Across 20 varieties of beans, lentils, chickpeas, and peas, this level of pulses corresponds to approximately ½ metric cups whole cooked pulses (mean: 105 ± 23 g—Pulse Canada unpublished data). A half cup is also comparable to the serving size of cooked pulses outlined in dietary guidelines from other developed regions including the United States (½ cup/serving) [9], Australia (75 g as a vegetable) [10], the United Kingdom (80 g/serving) [14], and Greece (100 g/serving) [16]. Furthermore, the Canadian Food Inspection Agency, the department that enforces Canada’s Food and Drug Regulations, has suggested that 100–200 mL represents a serving of pulses, which encompasses the 100 g daily target amount described herein [39]. Based on this information, 100 g/day, or ½ cup, is a realistic target for pulse consumption for Canadians and is a level of cooked pulses that can also be reasonably consumed in a single dose in isolation or combined with other foods such as cereals, vegetables, and pasta. Given these data collectively, for this analysis, 100 g/day cooked whole pulses (125 mL or ½ metric cups) was used as the target level of consumption for Canadian men and women ≥18 years of age [40].

Table 1 summarizes the input parameters corresponding to the model for T2D and CVD.

### 2.2. Step 1: Dietary Intake Success Rate

The first step of the analysis used consumption and frequency data to estimate the proportions of prospective Canadian consumers who are likely to include pulses in their habitual diets. Although pulses are recommended as part of a healthy diet by government agencies in Western countries, their consumption remains quite low. Specific to North America, an average of only 7.9% to 13.1% of the populace has been estimated to consume pulses on any given day [18,45], with a median intake level of 0.2 servings daily in the United States [46]. In Canada, using cross-sectional data (*n* = 20,156) from the 2004 Canadian Community Health Survey, Mudryj et al. [18] examined the prevalence of pulse consumption in adults (≥19 years old) and found that only 13% of Canadians consumed pulses on any given day, with mean intakes of 120 and 105 g/day for men and women, respectively. Additionally, a 2009 independent online survey-based marketing research and focus groups involving 1100 Canadian households revealed that 60% of Canadians consumed pulses one to three times per month and 20% consumed at least one type of pulse per week. Another 20% were considered “non-consumers” of pulses, when pulses were not consumed at home or at a restaurant over the preceded six months [38]. 

Given these observations, a sensitivity analysis of very pessimistic, pessimistic, optimistic, and very optimistic was established. The very optimistic scenario, representing a long-term shift in the dietary habits of populations, assumed an increase in pulse consumption equivalent to 50% of Canadians incorporating 100 g/day pulses into their diets to prevent an increase in CVD risk factors or, for patients with T2D, to use pulses to assist in adopting a low GI or high fiber dietary pattern to prevent T2D-related risk factors. The optimistic scenario assumed a 25% success rate, reflecting a medium-to-long-term estimate of financial savings through increased dietary pulse consumption. The pessimistic scenario hypothesized a 15% success rate to represent a less positive but more practical short-to-medium-term estimate of savings. Lastly, the very pessimistic scenario was set at 5% to estimate the immediate impact on the costs in a scenario where the success rate is extremely low and a small proportion of Canadians with and without T2D use pulses to facilitate dietary changes.

### 2.3. Step 2: Biomarker Reduction Rate

The second step of the model determined rates of reduction in recognized biomarkers of DM and CVD that are indirectly, through low GI or high fiber diets that include pulses, and directly associated with pulse intake (Table 1). Findings of meta-analyses were applied [27,33,34].

Based on the HbA1c concentrations, the prevalence of undiagnosed and diagnosed DM in Canada is 7.55% [47]. In a 2009 meta-analysis of 41 randomized controlled trials (RCTs) of diabetic and non-diabetic individuals, Sievenpiper et al. [27] estimated the effect of non-oil-seed pulses on glycemic control. There, standardized mean difference reductions in glycosylated blood proteins, measured as HbA1c or fructosamine concentrations, corresponded to an absolute reduction in HbA1c of ~0.48% with low GI or high fiber background diets that included pulses. Given that pulses are widely accepted as low GI and high fiber foods, dietary pulses can be used to help patients with DM manage HbA1c concentrations by adopting a low GI/ high fiber dietary pattern. The Food and Drug Administration has suggested that reductions in HbA1c of ≥0.3% are clinically significant [48].

For the CVD component, meta-analytic estimates of the absolute reductions in LDL-C concentrations and SBP, two well-known markers of CVD, following pulse intake were applied. Ha et al. [34] identified 26 RCTs of individuals with hyperlipidemia, normal lipid profiles, or a combination of both, and showed a mean difference of −0.17 mmol/L (95% CI −0.25 to −0.09 mmol/L), or 5% in LDL-C concentrations, with diets emphasizing pulse intake at a median dose of 130 g/day, compared to control diets. Another meta-analysis of 8 randomized isocaloric trials by Jayalath et al. [33] quantified the effects of dietary pulse intakes on blood pressure in people with and without hypertension. Following an average dose of 162 g/day of cooked pulses that were exchanged isocalorically for other foods, the authors showed a mean difference of −2.25 mm Hg (95% CI −4.22 to −0.28) in SBP. Both meta-analyses evaluated the effects of dose (<100 g/day vs. ≥100 g/day). Analysis of dichotomous variables demonstrated that the mean differences in LDL-C concentrations (−0.18 mmol/L) for studies that provided ≥100 g/day pulses aligned with the pooled effect of −0.17 mmol/L in Ha et al. [34]. A mean difference of −0.08 mmol/L in LDL-C was observed for studies that provided <100 g/day. Similarly, for Jayalath et al. [33], the mean difference in SBP for studies that provided ≥100 g/day was −2.89 mmHg compared to –2.25 mmHg for the pooled mean difference. For studies that provided <100 g/day, the effect on SBP was +1.60 mmHg. In both Ha et al. [34] and Jayalath et al. [33], the pooled effect was slightly below mean differences observed for studies that provided ≥100 g/day. Therefore, to integrate a more conservative approach to the present analysis, the pooled effect was used as the outcome for the effect of pulses on LDL-C concentrations and SBP. Similar to the biomarker reduction rate for HbA1c, given that the median and mean doses for both LDL-C and SBP exceeded 100 g/day, a proportional reduction in risk factors (per g) was modeled. Accordingly, −3.8% and −1.4 mmHg were used as the biomarker reduction rates, respectively. 

These recent meta-analytic findings were judged as the best method to decipher the relationship between pulses and well-recognized biomarkers of T2D and CVD in humans.

### 2.4. Step 3: Disease Incidence and Complication Reduction Rate

Given the paucity of direct or “hard endpoint” evidence of the association between low GI or high fiber diets and pulse consumption on incidence of complications from T2D and new cases of CVD, respectively, assumptions were made regarding the rate at which reductions in individual biomarkers related to DM and CVD would separately decrease by dietary patterns that include pulses. After examination of the relevant English-language medical and nutritional literature, assumptions for Step 3 were generated based on prospective observational and systematic meta-analytic studies [41,42,44]. Inclusion criteria and quality assessment are included in the studies of choice.

Although healthcare costs in Canada are broadly classified as DM, this study focused on the effects of dietary modification on outcomes in T2D patients. The inverse relationship between HbA1c concentrations and risk of complications from T2D was estimated based on the observational findings of Stratton et al. [41]. Among people with T2D, each 1% between-treatment absolute value difference in HbA1c was associated with 11–21% (an average of 16%) lower risk of any endpoint related to T2D between baseline and 10 years of follow up.

Estimated reductions in CVD risk following lower LDL-C concentrations were derived from a meta-analysis of randomized trials with statin therapy in people at low risk of vascular disease by the Cholesterol Treatment Trialists’ Collaborators [42] and a meta-regression analysis of randomized controlled cholesterol-lowering trials, in individuals with a history of CVD, with a moderate-risk of CVD, or with a high-risk of CVD, by Robinson et al. [43]. Both studies suggested that each 1% reduction in LDL-C concentrations was associated with an approximate 1% reduction in mortality due to cardiovascular events. These data complement trends from previous meta-analyses [49,50], where each 1% reduction in serum cholesterol concentrations corresponded to 2.5% and 4% reductions in mortality from CVD over the long term. Independently, CVD reduction following a decrease in blood pressure was estimated based on the meta-analytic findings of 147 randomized trials (*n* = 22,000) that evaluated the association between hypertensive medications and coronary heart disease (CHD) in participants with no history of vascular disease, a history of CHD, or a history of stroke [44]. Every 10 mm Hg reduction in SBP was associated with 22% reduction in CHD events [44]. This also complements the findings of a recent systematic review and meta-analysis of 123 randomized controlled blood pressure lowering trials (*n* = 613,815), where every 10 mm Hg reduction in SBP was shown to reduce the risk of major CVD events by 20% and CHD by 17% [51].

Calculations in this step suggested that low GI or high fiber diets, that include pulses, could decrease complications associated with T2D by 7.7% and that 100 g/day intake of pulses could decrease the incidence of CVD by 6.9% (Table 1). For the purpose of this model, it was assumed that the relative decrease in CVD risk factors per 100 g/day pulses corresponds to a decrease in the population-wide incidence of CVD of the same magnitude.

### 2.5. Step 4: Disease Cost Savings

The final step of this model assessed the potential annual cost savings that would accrue to the Canadian healthcare system and society following improvements in the respective management of T2D and rate of CVD. In Canada, costs of illnesses are typically broken down into direct and indirect categories. The former includes expenditures related to hospital care, physician care, and drugs, whereas the latter refers to the dollar value associated with lost production due to illness, injury, or premature death (i.e., morbidity and mortality costs) [52]. An overview of the most recent cost estimates associated with DM and CVD in Canada [52] and each of its provinces/territories [53] is summarized in Table 2 and Table 3, respectively, and has been discussed in detail elsewhere [54]. It is worthwhile to note that essential hypertension makes up over one quarter of all CVD costs in Canada (Supplementary Tables S1 and S2). As the outlined comprehensive estimates of DM and CVD costs were reported in year 2008 dollars [52], these values were inflated to their 2015 equivalent levels using Statistics Canada’s Consumer Price Index (health and personal care sub-index) [55] (Table 2). Furthermore, given that the majority (~90%) of diabetes cases are T2D [56], all costs linked to DM were assumed to be for T2D. The monetary values were analyzed according to previously described methods [54] to determine the reduction in disease costs that corresponds to each 1% decrease in endpoints associated with T2D and incidence of CVD in Canada (Table 4). 

In brief, components of the direct and indirect cost categories were assessed individually, where a 1% decline in disease incidence or complications was assumed to translate to a 1% reduction in cost estimates (1:1 ratio) for all components (i.e., physician care, drug mortality, and morbidity) except for the hospital care (1:0.25 ratio) (Table 4). Hospitalization costs include fixed and variable components. The fixed costs are those related to operating a hospital and will be incurred regardless of the disease prevalence. On the contrary, the variable costs are affected by the number of hospital admissions. In short-term hospital cost analyses [57,58,59], approximately 55% to 84% of hospital costs were previously estimated as fixed (i.e., salaries, buildings, and equipment) and 16% to 45% as variable (i.e., medications and supplies). Accordingly, for the purpose of this research, we assumed that declines in T2D endpoints and incidence of CVD would facilitate proportional reductions in the variable but not the fixed hospital-related costs. In this regard, the reduction in disease could lead to a reduced need for hospital capacity where fixed costs of factors such as salaries and buildings are able to adjust. Therefore, over the long term, this approach is conservative in nature. Costs of other components of the direct and indirect categories were assumed to decline in proportion to CVD incidence reductions, based on the assumption that fewer individuals with the disease would mean less physician visits, and, hence, less billings, less medication for treatment, and lower rates of premature mortality and morbidity due to the disease. The same assumptions were made for the reduction in endpoints from improved management of T2D with low GI or high fiber diets that include pulses. However, for analysis of T2D cost savings, physician costs remained unchanged as it was assumed that, despite a reduction in endpoints, patients with T2D would continue to visit their physician on an ongoing basis.

Additionally, in Canada, the delivery and administration of healthcare services is a provincial responsibility. The same approach (as described above) was thus also utilized to estimate the potential cost savings following intake of pulses in each Canadian province and territory, and takes into consideration the between-province differences in cost inputs [60].

For CVD-related incidence costs, although a 100 g/day pulses was used as the target consumption level to assess health and productivity-related cost reductions, it is acknowledged that actual consumption levels of pulses are widely distributed. Therefore, given the linearity of our model, the 100 g/day target for pulses intake and equivalent healthcare and productivity costs within each scenario can be scaled to a wider adoption range of pulse consumption.

### 2.6. Discounted Rate

The “very pessimistic” through to the “very optimistic” sensitivity analysis represents the short- (or immediate) to-long-term success rate for T2D patients adopting a low GI or high fiber diet that includes pulses, and preventing elevated LDL-C concentrations and SBP among Canadian adults that consume 100 g/day pulses. Accordingly, it is unlikely that the healthcare cost savings that correspond to success rates greater than 5% in the very pessimistic scenario would be realized in the present day. To determine the present value of cost-savings from the short-to-long term success rates (pessimistic to very optimistic scenarios) of incorporating pulses, a real discount rate of 8% was applied to the data using the following equation:

$$Discounted\;cost\;savings=savings\;at\;year\;0\;x\;\frac{1}{{\left(1+r\right)}^{n}}$$

where *r* = real discount rate (8%) and *n* = years into the future. The real discount rate of 8% aligns with the suggested rate from Health Canada [61]. Previous cost-benefit and impact analyses have used 20 years as the timeline for modeling the trajectory of healthcare costs over time [61,62,63]. For this dataset, the real discount rate was applied to present day savings for CVD and T2D healthcare savings at five-year increments for 20 years after 2015 (year 0): 0–4 years (very pessimistic), 5–9 years (pessimistic), 10–14 years (optimistic), and 15–19 years (very optimistic).

## 3. Results

Potential healthcare and productivity cost savings associated the use of pulses to decrease with T2D complications and CVD incidence reductions among 5%, 15%, 25%, and 50% of Canadian adult population (≥18 years of age) (*n* = 28,895,209 as of July 2015) [40], are summarized in Table 5 and Table 6.

Under the very optimistic scenario, assuming a 50% success rate and potential benefit over the long term, our analysis predicted total annual healthcare and related cost savings equal to Can$62.4 (95% CI $26.0–$98.8) million for T2D and Can$315.5 (95% CI $110.6–$520.4) million for CVD. The optimistic scenario, assuming a 25% success rate and medium-to-long-term benefit, predicted savings of Can$31.2 (95% CI $13.0–$49.4) million in T2D costs and Can$157.7 (95% CI $55.3–$260.2) million in CVD costs annually. With a 15% success rate and a more practical short-to-medium-term benefit, the pessimistic scenario showed savings of Can$18.7 (95% CI $7.8–$29.6) million in costs of T2D and Can$94.6 (95% CI $33.2–$156.1) million in costs of CVD annually. Finally, the very pessimistic scenario of a 5% success rate suggested total annual savings of Can$6.2 (95% CI $2.6–$9.9) million for T2D and Can$31.6 (95% CI $11.1–$52.0) million for CVD costs.

Table 7 and Table 8 summarize the potential financial savings in T2D and CVD total costs, respectively, by province/territory following the increased intakes of pulses. For T2D, total annual savings ranged from less than Can$0.1 (95% CI <$0.1–<$0.1) million in each of The Northwest Territories, Nunavut, and Yukon, in very pessimistic through very optimistic scenarios, to Can$2.4 (95% CI $1.0–$3.8) and Can$23.9 (95% CI $10.0–$37.9) million in very pessimistic and very optimistic scenarios, respectively, in Ontario; with other provincial cost saving figures falling in between (Table 7). Similarly, annual savings in CVD costs were estimated to range between less than Can$0.1 (95% CI <$0.1–<$0.1) million (very pessimistic) to Can$0.2 (95% CI $0.1–$0.4) million (very optimistic) in each of The Northwest Territories, Nunavut, and Yukon, and between Can$11.1 (95% CI $3.9–$18.3) and Can$111.3 (95% CI $39–$183.6) million in Ontario, given the very pessimistic through very optimistic scenarios (Table 8). Detailed estimates of savings in the costs of the diseases of interest within each province/territory following a low GI or high fiber diet that includes pulses, for T2D management, or one serving of 100 g/day of dietary pulse, for CVD risk reduction, are provided in Supplementary Tables S3–S15 for T2D and Supplementary Tables S16–S28 for CVD. Adult populations (≥18 years of age) in Canadian provinces, as of July 2015, were as follows: Alberta (~3,271,000), British Columbia (~3,853,000), Manitoba (~1,003,000), New Brunswick (~620,000), Newfoundland and Labrador (~437,000), Northwest Territories (~33,000), Nova Scotia (~780,000), Nunavut (~23,000), Ontario (~11,117,000), Prince Edward Island (~119,000), Quebec (~6,734,000), Saskatchewan (~875,000), and Yukon (~30,000) [40].

To account for time preference, the present values of adopting dietary paradigms of increasing success rates over a 20 year period are summarized in Table 9. At an 8% real discount rate, adopting a low GI or high fiber dietary pattern that includes pulses to facilitate lower HbA1c concentrations decreased the potential total health costs (direct and indirect) associated with better management of T2D by Can$228.9 (95% CI $95.4–$362.4) million. Over the same time frame, increasing the success rate of consuming 100 g/day pulses to facilitate concurrent reductions in LDL-C concentrations and SBP decreased the associated healthcare costs by Can$1158 (95% CI $406.1–$1909) million.

## 4. Discussion

Using a cost-of-illness approach and conservative estimates of risk factor reduction, results of this research suggest opportunities for significant savings in T2D and CVD costs in Canada when a reasonable daily serving of dietary pulses is consumed. Specifically, if 50% of the Canadian adult population increased their intake of pulses, over Can$370 (95% CI $137–$619) million could potentially be saved in T2D and CVD related healthcare and loss of productivity costs annually. Less optimistically, Can$38 (95% CI $14–$62) to Can$189 (95% CI $68–$310) million in annual cost savings in T2D and CVD could be realized if between 5% and 25% of Canadians used pulses to help manage T2D-related complications or prevent increases in LDL-C concentrations and SBP, respectively. Subgroup analyses of provincial and territorial expenditures demonstrated that the costs of T2D and CVD are substantial across Canada and each region stands to realize the relevant financial savings from enhanced consumption of pulses. Healthcare and related expenditures account for roughly 50% of provincial total spending. For example, the “Health, Healthy Living, and Seniors” portfolio accounted for Can$5.5 million out of a total budget of Can$11.9 million in the province of Manitoba in 2015 [64].

Evidence highlights that consistent consumption of healthy foods, such as pulse crops, can decrease risk factors for chronic diseases, which are of enormous burden on health and the economy. It is thus logical that increasing intakes of pulses could result in financial benefits within the global healthcare sector. To our knowledge, to date, no studies have examined this association. Data from this study suggest that when the effects of healthy dietary constituents are applied to a financial model, Canada, and perhaps other jurisdictions, warrant the implementation of preventative strategies and policies from legislators that promote healthy dietary patterns, and ensure healthcare remains affordable and productively maximized.

Results of this study make a compelling case for increasing the use of pulses within the dietary patterns of Canadians. Provincial and federal governments would welcome any alterations in lifestyle that reduces financial pressure within the publicly funded healthcare system in Canada. In addition, positive lifestyle choices, which can include increasing consumption of pulses, could potentially reduce the level of monetary support required for the management of T2D and CVD in Canada, and allow for available funds to be reallocated to the treatment of ailments unrelated to these diseases, improvements in the healthcare system, or other priorities, such as education and infrastructure. Societal benefits from reduced disease prevalence could also extend beyond specific expenditures related to health, and can encourage economic growth by increasing productivity and the formation of human and financial capital [65]. This is supported by the observed decrease in costs related to loss of productivity from T2D and CVD reported in this model as the rate of pulse consumption increases. At the same time, it cannot be overlooked that, over the long term, associated healthcare savings from increasing dietary intakes of pulses, or any other lifestyle related modifications, may not completely offset other possible healthcare and societal (i.e., pensions) expenditures that are linked to a longer lifespan. This has been demonstrated by van Baal et al. [66], where lifetime health expenditures were greatest among healthy non-smokers, intermediate for obese individuals, and lowest for smokers. However, such evidence is still limited and in addition to healthcare expenditures, an increase in quality of life is also a compelling benefit for undertaking strategies that reduce disease risk factors related to lifestyle. Deficits in health have been linked to reductions in quality of life outcomes [67] and serves as another driver for undertaking strategies, such as diet and exercise that promote longevity and prevent premature mortality. At >200 billion Canadian dollars per annum, healthcare will always represent a significant proportion of the Canadian government’s budgetary expenditures [52]. Thus, the identification and implementation of lifestyle choices that help to decrease premature mortality by preventing or delaying the onset of associated diseases, such as T2D and CVD, are unlikely to relieve all of the financial pressures that Canada’s healthcare system faces. However, these preventative measures are one of many elements that can assist with resource reallocation, and improve efficiency and value of Canadian healthcare, as well as help to prolong quality of life for its population.

Keeping inflation rates constant, it is important to acknowledge that the money has greater value in the present day because the return on investment into healthcare is immediate. To account for time preference, Table 9 demonstrated that, with an 8% real discount rate over a 20 year period, when success rates improve in five year increments, healthcare costs from the potential effects of pulses on CVD risk factors and low GI or high fiber diets on reduced T2D-related complications are higher compared to maintaining a constant success rate of 15% over the same time period. However, although an 8% discount rate aligns with Health Canada’s Cost-Benefit Analysis Guide [61], its relatively high rate could predict a lower than expected reduction in healthcare expenditures for CVD and T2D from an increase in pulse consumption in Canada. In the US, regulatory impact analyses typically use 3% and 7% discount rates to inform regulatory decisions [63]. That being said, given the uncertainty in increasing success rates around pulse consumption, 8% serves as a more conservative approach to considering time preference in this analysis. 

Before such financial benefits from positive shifts in intakes of pulses can happen, challenges that impede pulse consumption within Canada should be addressed. Although pulses and other legumes are promoted as part of a healthy diet by Canada’s Food Guide, adequate pulse consumption in Canada is low [18]. To increase consumption of pulses among Canadians, and perhaps other regions, initiatives such as government policies or programs that aggressively promote pulses as nutritious constituents of diets could be of assistance. For example, increased prominence and positive positioning of pulses in Canada’s dietary guidelines could help to enhance their familiarity and consumption among Canadians. In Canada’s Food Guide [4], pulses are identified as an “alternative” source of protein, a description that could be perceived as a negative attribute. Moving forward, healthy foods, such pulses, should be promoted in dietary guidelines without descriptions that may overshadow their nutritional value as well as historical prominence in human diets. In addition, given their nutritional composition, pulses could be identified as foods that are part of both the protein and fruit and vegetable food groups, which accords with how pulses are positioned in other regions such as the US [9] and Australia [10]. Given their composition, pulses could be promoted in same fashion as whole grains and cereals, which have also been demonstrated in dietary guidelines for China [68]. Interestingly, to help facilitate an increase in consumption, the French Agency for Food, Environmental and Occupational Health & Safety recently published the opinion that pulses should be an independent food group in the next iteration of France’s food-based dietary guidelines [69]. Increased use of pulses in nutritional policies in schools could also help to familiarize Canadians with pulses from a young age. The abovementioned strategies could help to improve the nutritional quality of Canadian diets. 

Education programs that target healthcare practitioners could encourage consumption of pulses to individuals already at risk of disease. In addition, there is a need for an increase in utilization of whole pulses and efficacious pulse-derived ingredients in the development of manufactured food products that will appeal to consumers. More fundamentally, consumer demand for such foods is necessary and will likely depend upon the success of educational initiatives. Approval of health claims and evidence-based messages around the healthful properties of pulses may help with manufacturers’ desire to incorporate and market the presence of new food products. It is hopeful that promotional efforts would increase the proportion of users beyond the levels assumed within the scenarios of this analysis.

In addition to promotional activities, increased use of whole pulses and pulse ingredients, such as flours and fiber fractions in familiar foods, can also enhance consumption of pulses and the nutrients that improve dietary quality and modulate disease risk factors of relevance. Research efforts continue to delineate improvements in protein quality of finished foods when cereals and pulses are used as complimentary amino acid profiles [70]. In addition, approaches to optimize the functionality of pulse-based flours could increase utilization by the food industry to bolster the nutrient density of commonly consumed foods [71,72,73,74,75].

While the potential effects of pulse consumption on incidence and cost reduction were separately analyzed for T2D and CVD, a decrease in endpoints associated with poor management of T2D could further reduce CVD-related expenditures. On its own, T2D is an independent risk factor for CVD [76] and pulses have been shown to reduce CVD risk factors in individuals with T2D [30]. Although T2D has been shown to increase the risk of CVD by two-to-four-fold [77], appropriate literature that quantifies the reduced risk in CVD from a decrease in incidence of T2D *per se* is, to our knowledge, lacking. At the same time, the use of cost estimates that are exclusive to CVD or T2D avoided double-counting and is a strength of this analysis. However, the widespread use of dietary patterns that include pulses to help drive financial benefits to Canada’s healthcare system could exceed the monetary savings reported in this analysis because of the possible benefits of pulses on other outcomes that affect healthcare resources, such as weight loss [78], gastrointestinal function [79], and nutrient deficiencies [80].

This work is, to our knowledge, the first to examine the potential savings in costs attributed to lower rates of CVD and management of T2D from adopting dietary patterns that include pulses in Canada and each of its provinces/territories. The present cost and saving estimates are, as such, expected to attract interest at both the provincial and federal decision-making levels. However, limitations are worth noting. First, while recent literature and national databases were reviewed for identification of consumer trends and healthcare-related costs, as with previous cost-of-illness analyses [54,81,82], isolating the effects of pulses from other elements of lifestyle is challenging.

Second, while rates of biomarker reduction were generated using the most recent aggregated effects on HbA1c, LDL-C, and SBP, a lack of dose response data examining the effects of pulses on CVD and T2D risk factors translated into a reliance on various assumptions that predicted effects per 100 g/day or low GI or high fiber diets that include pulses. This warrants deeper exploration of the association between pulse consumption and disease risk and complications. Furthermore, effects of pulses on costs associated with T2D were contingent on adopting a low GI or high fiber diet. Given, that pulses are an inherently low GI and high fiber food, it was reasonable that studies evaluating the effects of low GI and high fiber diets used pulses in establishing the corresponding dietary pattern. However, if a low GI or high fiber diet is a treatment or management strategy for T2D patients, other foods besides pulses with the same properties could be equally as effective in lowering HbA1c. On a related note, given that CVD is a broad category which includes diseases that may not be strongly associated with LDL-C or SBP changes, the reliance on the inherently higher costs of CVD than CHD in the present work, and, hence, estimations of the associated cost savings, may be perceived by some as a limitation. Alternatively, risks of conditions such as cerebrovascular disease, renal disease, and peripheral artery disease can also be linked to atherosclerosis due to higher LDL-C concentrations and/or SBP.

Third, substantial changes to Canadians’ dietary habits, especially among those who consume little to no pulses, would be needed to reach the 100 g/day benchmark used in this study to assess effects on CVD-related healthcare and societal costs. Although whole pulses have been shown to significantly decrease risk factors for CVD, for some, dosages of pulses required to impose a benefit could be substantial; even though 100 g/day of beans, peas, chickpeas, or lentils is well within the serving size guidance for pulses in many jurisdictions, including Canada [8,9,10,11,12,13,14,15,16]. The perceived association between consumption of legumes and gastrointestinal discomfort, such as bloating and flatulence, could also be a deterrent -although studies demonstrate that perceived discomfort with ≥100 g cooked pulses was marginal when consumed more than or equal to three weeks [83]. These challenges corresponding to adoption are further compounded by the fact that the diets of many Canadians encompass a variety of foods, making achievement of the recommended daily intake of pulses difficult. That being said, considering the results of this analysis that correspond to whole cooked pulses and the observation that daily servings of whole pulses already align with 100 g, increasing success rates among Canadians may rely more so on advocating for consumption, rather than dosage. Furthermore, recent consumer research showing increased awareness and desire for plant-based foods and meat alternatives [84,85] could help to drive increased intakes of pulses, especially over the long term. Given the low consumption rates in Canada, strategies that communicate an “eat as much as you can” approach may also be effective and could produce dietary benefits outside the scope T2D and CVD risk, such as improvements in dietary quality.

In addition, the incorporation of a variety of dietary adjustments could reduce risk factors for CVD and complications associated with T2D on an ongoing basis. Specifically, rather than taking a reductionist approach to dietary changes that focus on single exchanges of foods or nutrients, addressing overall quality of Canadian diets by promoting healthy and palatable dietary patterns is likely a more feasible strategy for long-term benefits to health and healthcare costs. These multifaceted dietary approaches may not necessarily require that efficacious foods be consumed on a daily basis. This has been demonstrated with the “portfolio diet” whereby the long term effectiveness of a dietary paradigm that incorporated almonds, viscous fibers, plant-based protein, and plant sterols substantially decreased LDL-C concentrations by 12% [86,87]. Under a dietary patterned approach, consumption of pulses a few times per week, alongside other risk factor-lowering foods, is likely a more appealing program.

In the end, it is important to emphasize that the current study is a modeling exercise to help elucidate the potential effects of diet on certain healthcare costs if pulses were consumed at increasing levels in Canada. We are mindful of the challenges for aligning Canadians to consuming pulses each day, no matter the amount. However, as previously discussed, this, and other studies that link diet to the financials associated with healthcare demonstrate the potentially favorable impact of positive lifestyle choices, beyond the individual, on societal healthcare resources. Future research including cost-benefit [88] and cost-effectiveness [89] analyses designed to assess the potential economic value of pulse intakes, compared to components of other dietary changes, is likely to draw further interest into this emerging arena of nutrition economics.

## 5. Conclusions

In conclusion, a substantial T2D and CVD related financial benefit for Canada is expected to accompany an improved health status with achievable levels of dietary pulse consumption. Given the extensive economic burden of the Canadian public healthcare system, any reductions in associated costs have important budgetary implications for society. Findings of the present work highlight an opportunity to facilitate significant health-related cost savings that could be applied to Canadian healthcare or re-assigned to other priority domains, following increased consumption of pulse crops. Whether these potential cost savings will be partly or completely offset by other healthcare costs associated with longevity and diseases of the elderly remains to be investigated over the long term.

## Figures and Tables

**Table 1:** Summary of the cost-of-illness analysis input parameters and corresponding sources.

Parameter	Men and Women	Source
Intake of pulses for the model, g/day ^1^	0	Mudryj et al. [18]
Target pulse intake (1 serving), g/day ^2^	100	Mudryj et al. [18]
Success rate (proportions of prospective consumers ^3^)	Very pessimistic (5%) Pessimistic (15%) Optimistic (25%) Very optimistic (50%)	Estimates
T2D		
Mean absolute reduction in HbA1c with low GI or high fiber diets that include pulses (95% CI)	−0.48% (0.20–0.76)	Sievenpiper et al. [27]
T2D complications risk reduction based on HbA1c lowering (95% CI) ^4^	−7.7% (3.2–12.2)	Stratton et al. [41]
CVD		
LDL-C percent reduction per 1 serving pulse intake (95% CI)	−3.8% (2.0–5.7)	Ha et al. [34]
CVD risk reduction based on LDL-C lowering (95% CI) ^5^	−3.8% (2.0–5.7)	Treatment Trialists‘ Collaborators [42]; Robinson et al. [43]
SBP absolute value reduction per 1 serving pulse intake (95% CI)	−1.4 mm Hg (0.2–2.6)	Jayalath et al. [33]
CVD risk reduction based on SBP lowering (95% CI) ^6^	−3.1% (0.4–5.7)	Law et al. [44]

^1^ Based on findings of Mudryj et al. [18], where on any given day 87% of Canadians reported no pulse consumption (0 g/day). CVD, cardiovascular disease; GI, glycemic index; HbA1c, glycated hemoglobin; LDL-C, LDL-cholesterol; SBP, systolic blood pressure; T2D, type 2 diabetes. ^2^ Consumption of 100 g/day was considered a reasonable level to adopt into Canadian dietary patterns for decreasing incidence of CVD risk factors. ^3^ Proportions of the Canadian adult population (≥18 years of age) who would consume a low GI or high fiber diet that includes pulses for T2D management or one serving of 100 g/day of dietary pulse for CVD risk reduction over short term (very pessimistic), short-to-medium term (pessimistic), medium-to-long term (optimistic), and long term (very optimistic) scenarios. Estimations are partly based on findings of Mudryi et al. [18] and an online survey-based marketing research by Ipsos Reid [38]. ^4^ Based on findings of Stratton et al. [41], where each 1% between-treatment difference in HbA1c was associated with 11–21% (average 16%) lower risk of any endpoint related to T2D between baseline and 10 years of follow up. ^5^ Based on findings of the Cholesterol Treatment Trialists’ Collaborators [42] and Robinson et al. [43], where each 1% reduction in LDL-C was associated with a 1% reduction in mortality due to cardiovascular events. ^6^ Based on findings of Law et al. [44], where each 10 mm Hg reduction in SBP was associated with 22% reduction in CVD events.

**Table 2:** Summary of diabetes and cardiovascular disease costs in Canada (Can$ million).

	2008 ^1^	2015 ^2^
*Direct costs*		
Hospital care		
Diabetes	492.7	545.7
CVD	5068	5613
Physician care		
Diabetes	487.3	539.7
CVD	2352	2605
Drug		
Diabetes	1198	1327
CVD	4273	4732
Total direct costs		
Diabetes	2178	2412
CVD	11,690	12,950
*Indirect costs* ^3^		
Due to mortality		
Diabetes	12.3	13.6
CVD	92.4	102.3
Due to morbidity		
Diabetes	132.9	147.2
CVD	269.6	298.6
Total indirect costs		
Diabetes	145.2	160.8
CVD	362.0	400.9
Total costs		
Diabetes	2323	2573
CVD	12,060	13,350

^1^ From the Economic Burden of Illness 2005–2008 report [52]. CVD, cardiovascular disease. ^2^ Current dollars based on adjustments of inflation rates according to Statistics Canada Consumer Price Index [55]. ^3^ Indirect costs only include values of lost production due to reduced working time associated with illness, injury, or premature death, and do not include any valuation of morbidity and mortality themselves.

**Table 3:** Summary of diabetes and cardiovascular disease total cost (direct + indirect) by province/territory in Canada (Can$ million) ^1^.

	Diabetes	CVD
Alberta	223.2	1177
British Columbia	283.1	1602
Manitoba	80.0	465.9
New Brunswick	59.2	308.7
Newfoundland and Labrador	43.1	236.7
Northwest Territories	2.8	13.1
Nova Scotia	71.7	397.6
Nunavut	1.1	7.2
Ontario	993.5	4660
Prince Edward Island	10.6	55.1
Quebec	460.2	3002
Saskatchewan	64.0	406.3
Yukon	0.8	6.4

^1^ From the EBIC Custom Report Generator 2008 data [53] with adjustments of inflation rates for year 2015 according to Statistics Canada Consumer Price Index [55]. CVD, cardiovascular disease.

**Table 4:** Estimations of percent cost reduction corresponding to each 1% decrease in incidence of each of type 2 diabetes and cardiovascular disease.

	% Reduction
*Direct costs*	
Hospital care ^1^	0.25
Physician care	1.0
Drug	1.0
*Indirect costs* ^2^	
Due to mortality	1.0
Due to morbidity	1.0

^1^ Average reduction based on findings of Roberts et al. [57], Williams [58], and Lave and Lave [59], which estimated that 16% to 45% of hospitalization costs are variable (i.e., medications and supplies) and 55% to 84% are fixed (i.e., salaries, buildings, and equipment). ^2^ Indirect costs only include values of lost production due to reduced working time associated with illness, injury, or premature death, and do not include any valuation of morbidity and mortality themselves.

**Table 5:** Potential annual savings in healthcare and related costs of type 2 diabetes among Canadian adults from low glycemic and/or high fiber diets that include pulses (Can$ million) ^1^.

	Scenario			
	Very Pessimistic	Pessimistic	Optimistic	Very Optimistic
*Direct cost savings*				
Hospital care	0.5 (0.2–0.8)	1.6 (0.7–2.5)	2.6 (1.1–4.1)	5.2 (2.2–8.3)
Physician care	0.0	0.0	0.0	0.0
Drug	5.1 (2.1–8.1)	15.3 (7.0–24.2)	25.5 (10.6–40.3)	51.0 (21.2–80.7)
**Total direct cost savings**	**5.6 (2.3–8.9)**	**16.9 (24.0–26.7)**	**28.1 (11.7–44.5)**	**56.2 (23.4–89.0)**
*Indirect cost savings* ^2^				
Due to mortality	0.1 (<0.1–0.1)	0.2 (0.1–0.2)	0.3 (0.1–0.4)	0.5 (0.2–0.8)
Due to morbidity	0.6 (0.2–0.9)	1.7 (0.7–2.7)	2.8 (1.2–4.5)	5.7 (2.4–8.9)
**Total indirect cost savings**	**0.6 (0.3–1.0)**	**1.9 (0.8–2.9)**	**3.1 (1.3–4.9)**	**6.2 (2.6–9.8)**
**Total cost savings**	**6.2 (2.6–9.9)**	**18.7 (7.8–29.6)**	**31.2 (13.0–49.4)**	**62.4 (26.0–98.8)**

^1^ Data (95% CI) represent type 2 diabetes-related financial savings following reduction in HbA1c concentrations with the adoption of a low GI or high fiber diet that includes pulses for men and women [4] (Table 1). Given that patients with T2D are expected to continue to visit their physicians, these costs remained unchanged. The very optimistic scenario is a long-term estimate of potential savings when 50% of Canadian adults (≥18 years of age) with T2D consume a low GI or high fiber diet with dietary pulses. The optimistic scenario is a medium-to-long-term pragmatic estimate of potential savings when 25% of adults in Canada with T2D use pulses to adopt a low GI or high fiber diet. The pessimistic and very pessimistic scenario is a practical short-to-medium-term, and immediate estimate of cost savings that could follow when 15% and 5% of adults with T2D follow a low GI or high fiber diet with pulses. ^2^ Indirect costs only include values of lost production due to reduced working time associated with illness, injury, or premature death, and do not include any valuation of morbidity and mortality themselves.

**Table 6:** Potential annual savings in healthcare and related costs of cardiovascular disease among Canadian adults from 100 g/day dietary pulse intake (Can$ million) ^1^.

	Scenario			
	Very Pessimistic	Pessimistic	Optimistic	Very Optimistic
Cost savings following LDL-C reduction				
*Direct cost savings*				
Hospital care	2.7 (1.4–4.0)	8.1 (4.3–11.9)	13.5 (7.21–9.8)	27.0 (14.3–39.7)
Physician care	5.0 (2.7–7.4)	15.0 (8.0–22.1)	25.0 (13.3–36.8)	50.1 (26.6–73.6)
Drug	9.1 (4.8–13.4)	27.3 (14.5–40.1)	45.5 (24.1–66.9)	91.0 (48.2–133.8)
**Total direct cost savings**	**16.8 (8.9–24.7)**	**50.4 (26.7–74.1)**	**84.0 (44.5–123.5)**	**168.1 (89.1–247.1)**
*Indirect cost savings* ^2^				
Due to mortality	0.2 (0.1–0.3)	0.6 (0.3–0.9)	1.0 (0.5–1.4)	2.0 (1.0–2.9)
Due to morbidity	0.6 (0.3–0.8)	1.7 (0.9–2.5)	2.9 (1.5–4.2)	5.7 (3.0–8.4)
**Total indirect cost savings**	**0.8 (0.4–1.1)**	**2.3 (1.2–3.4)**	**3.9 (2.0–5.7)**	**7.7 (4.1–11.3)**
**Total cost savings**	**17.6 (9.3–25.8)**	**52.7 (28.0–77.5)**	**87.9 (46.6–129.2)**	**175.8 (93.2–258.4)**
**Cost savings following SBP reduction**				
*Direct cost savings*				
Hospital care	2.1 (0.3–4.0)	6.4 (0.8–12.1)	10.7 (1.3–20.1)	21.4 (2.7–40.2)
Physician care	4.0 (0.5–7.5)	11.9 (1.5–22.4)	19.9 (2.5–37.3)	39.8 (5.0–74.6)
Drug	7.2 (0.9–13.6)	21.7 (2.7–40.7)	36.1 (4.5–67.8)	72.3 (9.0–135.6)
**Total direct cost savings**	**13.4 (1.7–25.0)**	**40.1 (5.0–75.1)**	**66.8 (8.3–125.2)**	**133.5 (16.6–250.4)**
*Indirect cost savings* ^2^				
Due to mortality	0.2 (<0.1–0.3)	0.5 (0.1–0.9)	0.8 (0.1–1.5)	1.6 (0.2–2.9)
Due to morbidity	0.5 (0.1–0.9)	1.4 (0.2–2.6)	2.3 (0.3–4.3)	4.6 (0.6–8.6)
**Total indirect cost savings**	**0.6 (0.1–1.1)**	**1.8 (0.2–3.4)**	**3.1 (0.4–5.7)**	**6.1 (0.8–11.5)**
**Total cost savings**	**14.0 (1.7–26.2)**	**41.9 (5.2–78.6)**	**69.8 (8.7–131.0)**	**139.7 (17.4–261.9)**
**Cost savings following LDL-C and SBP reduction combined**				
*Direct cost savings*				
Hospital care	4.8 (1.7–8.0)	14.5 (5.1–24.0)	24.2 (39.9–8.5)	48.4 (17.0–79.9)
Physician care	9.0 (3.2–14.8)	26.9 (9.5–44.5)	44.9 (15.8–74.1)	89.9 (31.5–148.3)
Drug	16.3 (5.7–26.9)	49.0 (17.2–80.8)	81.6 (28.6–134.7)	163.3 (57.2–269.4)
**Total direct cost savings**	**30.2 (10.6–49.8)**	**90.5 (31.7–149.3)**	**150.8 (52.9–248.8)**	**301.6 (105.7–497.5)**
*Indirect cost savings* ^2^				
Due to mortality	0.4 (0.1–0.6)	1.1 (0.4–1.7)	1.8 (0.6–2.9)	3.6 (1.2–5.8)
Due to morbidity	1.1 (0.4–1.7)	3.1 (1.1–5.1)	5.2 (1.8–8.5)	10.3 (3.6–17.0)
**Total indirect cost savings**	**1.4 (0.5–2.3)**	**4.1 (1.5–6.8)**	**7.0 (2.4–11.4)**	**13.8 (4.8–22.8)**
**Total cost savings**	**31.6 (11.1–52.0)**	**94.6 (33.2–156.1)**	**157.7 (55.3–260.2)**	**315.5 (110.6–520.4)**

^1^ Data (95% CI) represent cardiovascular disease-related financial savings following reductions in LDL-cholesterol concentrations and systolic blood pressure with the consumption of 100 g/day pulses for men and women [4] (Table 1). The very optimistic scenario is an estimate of potential savings when 50% of Canadian adults (≥18 years of age) consume one 100 g/day serving of dietary pulses. The optimistic scenario is a medium-to-long-term pragmatic estimate of potential savings when 25% of adults in Canada consume pulse regularly. The pessimistic scenario is a practical short-to-medium-term estimate of cost savings that could follow the dietary pulse consumptions among 15% of adults. The very pessimistic scenario is an immediate estimate when 5% of Canadian adults adopt 100 g/day serving of pulses. LDL-C, LDL-cholesterol; SBP, systolic blood pressure. ^2^ Indirect costs only include values of lost production due to reduced working time associated with illness, injury, or premature death, and do not include any valuation of morbidity and mortality themselves.

**Table 7:** Sum of potential annual savings in healthcare and related costs of type 2 diabetes among Canadian adults from low glycemic and/or high fiber diets that include pulses by province/territory (Can$ million) ^1^.

	Scenario			
	Very Pessimistic	Pessimistic	Optimistic	Very Optimistic
Alberta	0.5 (0.2–0.8)	1.5 (0.6–2.3)	2.4 (1.0–3.8)	4.9 (2.0–7.7)
British Columbia	0.5 (0.2–0.8)	1.5 (0.6–2.4)	2.6 (1.1–4.1)	5.1 (2.1–8.1)
Manitoba	0.2 (0.1–0.3)	0.5 (0.2–0.8)	0.8 (0.3–1.3)	1.7 (0.7–2.6)
New Brunswick	0.1 (0.1–0.2)	0.4 (0.2–0.6)	0.7 (0.3–1.1)	1.3 (0.6–2.1)
Newfoundland and Labrador	0.1 (<0.1–0.2)	0.3 (0.1–0.5)	0.5 (0.2–0.8)	1.0 (0.4–1.6)
Northwest Territories	<0.1 (<0.1–<0.1)	<0.1 (<0.1–<0.1)	<0.1 (<0.1–<0.1)	<0.1 (<0.1–<0.1)
Nova Scotia	0.2 (0.1–0.3)	0.5 (0.2–0.8)	0.8 (0.3–1.3)	1.7 (0.7–2.6)
Nunavut	<0.1 (<0.1–<0.1)	<0.1 (<0.1–<0.1)	<0.1 (<0.1–<0.1)	<0.1 (<0.1–<0.1)
Ontario	2.4 (1.0–3.8)	7.2 (3.0–11.4)	12.0 (5.0–18.9)	23.9 (10.0–37.9)
Prince Edward Island	<0.1 (<0.1–<0.1)	0.1 (<0.1–0.1)	0.1 (<0.1–0.2)	0.2 (0.1–0.4)
Quebec	1.2 (0.5–2.0)	3.7 (1.5–5.9)	6.2 (2.6–9.8)	12.3 (5.1–19.5)
Saskatchewan	0.1 (0.1–0.2)	0.4 (0.2–0.7)	0.7 (0.3–1.1)	1.4 (0.6–2.3)
Yukon	<0.1 (<0.1–<0.1)	<0.1 (<0.1–<0.1)	<0.1 (<0.1–<0.1)	<0.1 (<0.1–<0.1)

^1^ Data (95% CI) represent type 2 diabetes-related financial savings following reduction in HbA1c concentrations with the adoption of a low GI or high fiber diet that includes pulses for men and women [4] (Table 1). The very optimistic scenario is a long-term estimate of potential savings when 50% of Canadian adults (≥18 years of age) with T2D consume a low GI or high fiber diet with dietary pulses. The optimistic scenario is a medium-to-long-term pragmatic estimate of potential savings when 25% of adults in Canada with T2D use pulses to adopt a low GI or high fiber diet. The pessimistic and very pessimistic scenario is a practical short-to-medium-term, and immediate estimate of cost savings that could follow when 15% and 5% of adults with T2D follow a low GI or high fiber diet with pulses.

**Table 8:** Sum of potential annual savings in healthcare and related costs of cardiovascular disease among Canadian adults from 100 g/day dietary pulse intake by province and territory (Can$ million).^1^

	Scenario			
	Very optimistic	Optimistic	Pessimistic	Very pessimistic
Alberta				
Following LDL-C reduction	15.7 (8.3–23.1)	7.9 (4.2–11.6)	4.7 (2.5–6.9)	1.6 (0.8–2.3)
Following SBP reduction	12.5 (1.6–23.4)	6.2 (0.8–11.7)	3.7 (0.5–7.0)	1.2 (0.2–2.3)
British Columbia				
Following LDL-C reduction	19.3 (10.2–28.3)	9.6(5.1–14.2)	5.8 (3.1–8.5)	1.9 (1.0–2.8)
Following SBP reduction	15.3 (1.9–28.7)	7.7 (1.0–14.3)	4.6 (0.6–8.6)	1.5 (0.2–2.9)
Manitoba				
Following LDL-C reduction	5.9 (3.1–8.6)	2.9 (1.6–4.3)	1.8 (0.9–2.6)	0.6 (0.3–0.9)
Following SBP reduction	4.7 (0.6–8.8)	2.3 (0.3–4.4)	1.4 (0.2–2.6)	0.5 (0.1–0.9)
New Brunswick				
Following LDL-C reduction	3.9 (2.0–5.7)	1.9 (1.0–2.8)	1.2 (0.6–1.7)	0.4 (0.2–0.6)
Following SBP reduction	3.1 (0.4–5.8)	1.5 (0.2–2.9)	0.9 (0.1–1.7)	0.3 (<0.1–0.6)
Newfoundland and Labrador				
Following LDL-C reduction	2.8 (1.5–4.2)	1.4 (0.8–2.1)	0.9 (0.5–1.3)	0.3 (0.2–0.4)
Following SBP reduction	2.3 (0.3–4.2)	1.1 (0.1–2.1)	0.7 (0.1–1.3)	0.2 (<0.1–0.4)
Northwest Territories				
Following LDL-C reduction	0.1 (0.1–0.2)	0.1 (<0.1–0.1)	<0.1 (<0.1–0.1)	<0.1 (<0.1-<0.1)
Following SBP reduction	0.1 (<0.1–0.2)	0.1 (<0.1–0.1)	<0.1 (<0.1–0.1)	<0.1 (<0.1-<0.1)
Nova Scotia				
Following LDL-C reduction	5.0 (2.6–7.3)	2.5 (1.3–3.6)	1.5 (0.8–2.2)	0.5 (0.3–0.7)
Following SBP reduction	3.9 (0.5–7.4)	2.0 (0.2–3.7)	1.2 (0.1–2.2)	0.4 (<0.1–0.7)
Nunavut				
Following LDL-C reduction	0.1 (0.1–0.1)	<0.1 (<0.1–0.1)	<0.1 (<0.1–<0.1)	<0.1 (<0.1–<0.1)
Following SBP reduction	0.1 (<0.1–0.1)	<0.1 (<0.1–0.1)	<0.1 (<0.1–<0.1)	<0.1 (<0.1–<0.1)
Ontario				
Following LDL-C reduction	62.0 (32.9–91.2)	31.0 (16.4–45.6)	18.6 (9.9–27.3)	6.2 (3.3–9.1)
Following SBP reduction	49.3 (6.1–92.4)	24.6 (3.1–46.2)	14.8 (1.8–27.7)	4.9 (0.6–9.2)
Prince Edward Island				
Following LDL-C reduction	0.7 (0.4–1.0)	0.3 (0.2–0.5)	0.2 (0.1–0.3)	0.1 (<0.1–0.1)
Following SBP reduction	0.6 (0.1–1.0)	0.3 (<0.1–0.5)	0.2 (<0.1–0.3)	0.1 (<0.1–0.1)
Quebec				
Following LDL-C reduction	40.1 (21.2–58.9)	20.0 (10.6–29.5)	12.0 (6.4–17.7)	4.0 (2.1–5.9)
Following SBP reduction	31.9 (4.0–59.7)	15.9 (2.0–29.9)	9.6 (1.2–17.9)	3.2 (0.4–6.0)
Saskatchewan				
Following LDL-C reduction	5.1 (2.7–7.5)	2.6 (1.4–3.8)	1.5 (0.8–2.3)	0.5 (0.3–0.8)
Following SBP reduction	4.1 (0.5–7.6)	2.0 (0.3–3.8)	1.2 (0.2–2.3)	0.4 (0.1–0.8)
Yukon				
Following LDL-C reduction	0.1 (<0.1–0.1)	<0.1 (<0.1–<0.1)	<0.1 (<0.1–<0.1)	<0.1 (<0.1–<0.1)
Following SBP reduction	0.1 (<0.1–0.1)	<0.1 (<0.1–<0.1)	<0.1 (<0.1–<0.1)	<0.1 (<0.1–<0.1)

^1^ Data (95% CI) represent cardiovascular disease-related financial savings following reductions in LDL-cholesterol concentrations and systolic blood pressure with the consumption of 100 g/day pulses for men and women [4] (Table 1). The very optimistic scenario is an estimate of potential savings when 50% of Canadian adults (≥18 years of age) consume one 100 g/day serving of dietary pulses. The optimistic scenario is a medium-to-long-term pragmatic estimate of potential savings when 25% of adults in Canada consume pulse regularly. The pessimistic scenario is a practical short-to-medium- term estimate of cost savings that could follow the dietary pulse consumptions among 15% of adults. The very pessimistic scenario is an immediate estimate when 5% of Canadian adults adopt 100 g/day serving of pulses. LDL-C, LDL-cholesterol; SBP, systolic blood pressure.

**Table 9:** Sum of potential total discounted savings on healthcare and related costs of type 2 diabetes and cardiovascular disease among Canadian adults from low glycemic and/or high fiber diets that include pulses or 100 g/day dietary pulse intake over short-term and long-term periods (Can$ million).^1^

	Scenario			
	Very Pessimistic	Pessimistic	Optimistic	Very Optimistic
*Years 0 to 4*				
Diabetes	**26.9 (11.2–42.6)**	80.7 (33.6–127.8)	134.5 (56.0–212.9)	269.0 (112.1–425.9)
CVD	**136.3 (47.9–224.2)**	407.9 (143.2–673.1)	680.0 (238.5–1122)	1361 (476.9–2244)
*Years 5 to 9*				
Diabetes	18.3 (7.6–29.0)	**54.9 (22.9–86.9)**	91.5 (38.1–144.9)	183.0 (76.3–289.8)
CVD	92.7 (32.6–152.6)	**277.6 (97.4–458.1)**	462.8 (162.3–763.6)	925.9 (324.6–1527)
*Years 10 to 14*				
Diabetes	12.5 (5.2–19.7)	37.4 (15.6–59.2)	**62.3 (26.0–98.6)**	124.6 (51.9–197.3)
CVD	63.1 (22.2–103.9)	188.9 (66.3–311.8)	**315.0 (110.5–519.7)**	630.2 (220.9–1039)
*Years 15 to 19*				
Diabetes	8.5 (3.5–13.4)	25.4 (10.6–40.3)	42.4 (17.7–67.1)	**84.8 (35.5–134.2)**
CVD	43.0 (15.1–70.7)	128.6 (45.1–212.2)	214.4 (75.2–353.7)	**428.9 (150.3–707.4)**
**Total discounted savings for each scenario (2015–2034)**				
**Diabetes**	**66.1 (27.6–104.7)**	**198.4 (82.7–314.2)**	**330.7 (137.8–523.6)**	**661.4 (275.6–1047)**
**CVD**	**335.1 (117.7–551.4)**	**1003 (352.0–1655)**	**1672 (568.4–2759)**	**3345 (1173–5518)**
**Total incremental discounted savings: adoption of each scenario every 5 years (2015–2034)**				
**Diabetes**	**-**	**-**	**-**	**228.9 (95.4–362.4)**
**CVD**	**-**	**-**	**-**	**1158 (406.1–1909)**

^1^ Data (95% CI) represent type 2 diabetes-related financial savings following reduction in HbA1c concentrations with adoption of a low GI or high fiber diet that includes pulses, and cardiovascular disease-related financial savings following reduction in LDL-cholesterol concentrations and systolic blood pressure with the consumption of 100 g/day pulses, for men and women. The very optimistic scenario is a long-term estimate of potential savings when 50% of Canadian adults (≥18 years of age) with or without T2D consume a low GI or high fiber diet with dietary pulses, or one 100 g/day serving of dietary pulses. The optimistic scenario is a medium-to-long-term pragmatic estimate of potential savings when 25% of adults in Canada consume pulse regularly. The pessimistic scenario is a practical short-to-medium-term estimate of cost savings that could follow the dietary pulse consumptions among 15% of adults. The very pessimistic scenario is an immediate estimate when 5% of Canadian adults with or without T2D follow a low GI or high fiber diet with pulses or 100 g/day serving of pulses. CVD, cardiovascular disease.

## Supplementary Materials

The followings are available online at http://www.mdpi.com/2072-6643/9/7/793/s1, Tables S1. Summary of essential hypertension costs in Canada (Can$ million) Table S2. Summary of essential hypertension total cost (direct + indirect) by province/territory in Canada (Can$ million). Table S3. Potential annual savings in healthcare and related costs of type 2 diabetes among Canadian adults from low glycemic and/or high fiber diets that include pulses in Alberta (Can$ million). Table S4. Potential annual savings in healthcare and related costs of type 2 diabetes among Canadian adults from low glycemic and/or high fiber diets that include pulses in British Columbia (Can$ million). Table S5. Potential annual savings in healthcare and related costs of type 2 diabetes among Canadian adults from low glycemic and/or high fiber diets that include pulses in Manitoba (Can$ million). Table S6. Potential annual savings in healthcare and related costs of type 2 diabetes among Canadian adults from low glycemic and/or high fiber diets that include pulses in New Brunswick (Can$ million). Table S7. Potential annual savings in healthcare and related costs of type 2 diabetes among Canadian adults from low glycemic and/or high fiber diets that include pulses in Newfoundland and Labrador (Can$ million). Table S8. Potential annual savings in healthcare and related costs of type 2 diabetes among Canadian adults from low glycemic and/or high fiber diets that include pulses in The Northwest Territories (Can$ million). Table S9. Potential annual savings in healthcare and related costs of type 2 diabetes among Canadian adults from low glycemic and/or high fiber diets that include pulses in Nova Scotia (Can$ million). Table S10. Potential annual savings in healthcare and related costs of type 2 diabetes among Canadian adults from low glycemic and/or high fiber diets that include pulses in Nunavut (Can$ million). Table S11. Potential annual savings in healthcare and related costs of type 2 diabetes among Canadian adults from low glycemic and/or high fiber diets that include pulses in Ontario (Can$ million). Table S12. Potential annual savings in healthcare and related costs of type 2 diabetes among Canadian adults from low glycemic and/or high fiber diets that include pulses in Prince Edward Island (Can$ million). Table S13. Potential annual savings in healthcare and related costs of type 2 diabetes among Canadian adults from low glycemic and/or high fiber diets that include pulses in Quebec (Can$ million). Table S14. Potential annual savings in healthcare and related costs of type 2 diabetes among Canadian adults from low glycemic and/or high fiber diets that include pulses in Saskatchewan (Can$ million). Table S15. Potential annual savings in healthcare and related costs of type 2 diabetes among Canadian adults from low glycemic and/or high fiber diets that include pulses in Yukon (Can$ million). Table S16. Potential annual savings in healthcare and related costs of cardiovascular disease among Canadian adults from 100 g/day dietary pulse intake in Alberta (Can$ million). Table S17. Potential annual savings in healthcare and related costs of cardiovascular disease among Canadian adults from 100 g/day dietary pulse intake in British Columbia (Can$ million). Table S18. Potential annual savings in healthcare and related costs of cardiovascular disease among Canadian adults from 100 g/day dietary pulse intake in Manitoba (Can$ million). Table S19. Potential annual savings in healthcare and related costs of cardiovascular disease among Canadian adults from 100 g/day dietary pulse intake in New Brunswick (Can$ million). Table S20. Potential annual savings in healthcare and related costs of cardiovascular disease among Canadian adults from 100 g/day dietary pulse intake in Newfoundland and Labrador (Can$ million). Table S21. Potential annual savings in healthcare and related costs of cardiovascular disease among Canadian adults from 100 g/day dietary pulse intake in The Northwest Territories (Can$ million). Table S22. Potential annual savings in healthcare and related costs of cardiovascular disease among Canadian adults from 100 g/day dietary pulse intake in Nova Scotia (Can$ million). Table S23. Potential annual savings in healthcare and related costs of cardiovascular disease among Canadian adults from 100 g/day dietary pulse intake in Nunavut (Can$ million). Table S24. Potential annual savings in healthcare and related costs of cardiovascular disease among Canadian adults from 100 g/day dietary pulse intake in Ontario (Can$ million). Table S25. Potential annual savings in healthcare and related costs of cardiovascular disease among Canadian adults from 100 g/day dietary pulse intake in Prince Edward Island (Can$ million). Table S26. Potential annual savings in healthcare and related costs of cardiovascular disease among Canadian adults from 100 g/day dietary pulse intake in Quebec (Can$ million). Table S27. Potential annual savings in healthcare and related costs of cardiovascular disease among Canadian adults from 100 g/day dietary pulse intake in Saskatchewan (Can $ million). Table S28. Potential annual savings in healthcare and related costs of cardiovascular disease among Canadian adults from 100 g/day dietary pulse intake in Yukon (Can$ million).
