# Supplementary material for: Canadian Potential Healthcare and Societal Cost Savings from Consumption of Pulses: A Cost-Of-Illness Analysis
Source: Nutrients. 2017 Jul 22;9(7):793. doi: 10.3390/nu9070793 (PMC5537906; doi:10.3390/nu9070793)
Supplement: Supplementary file 1 [file nutrients-09-00793-s001.pdf]

Supplementary Materials: Canadian potential healthcare and societal cost savings from consumption of pulses: a cost-of-illness analysis

Mohammad M.H. Abdullah, Christopher P.F. Marinangeli, Peter J.H. Jones, Jared G. Carlberg

Table S1. Summary of essential hypertension costs in Canada (Can $ million).

	**2008**[1]	**2015**[2]
Direct costs		
Hospital	38.2	40.0
Physician care	679.4	711.3
Drug	2,512	2,629
**Total direct costs**	**3,229**	**3,381**
Indirect costs[3]		
Due to Mortality	0.6	0.6
Due to Morbidity	NA	NA
**Total indirect costs**	**0.6**	**0.6**
**Total costs**	**3,230**	**3,381**

[1] From the EBIC Custom Report Generator 2008 data [1] with adjustments of inflation rates for year 2015 according to Statistics Canada Consumer Price Index [2]. NA, not available. [2] Current dollars based on adjustments of inflation rates according to Statistics Canada Consumer Price Index [2]. [3] Indirect costs only include values of lost production due to reduced working time associated with illness, injury, or premature death, and do not include any valuation of morbidity and mortality themselves.

Table S2. Summary of essential hypertension total cost (direct + indirect) by province/territory in Canada (Can $ million).[1]

	**Essential hypertension**
Alberta	318.4
British Columbia	393.3
Manitoba	121.3
New Brunswick	89.0
Newfoundland and Labrador	66.5
Northwest Territories	2.3
Nova Scotia	116.1
Nunavut	2.0
Ontario	1,344
Prince Edward Island	16.8
Quebec	801.2
Saskatchewan	109.1
Yukon	1.2

[1] From the EBIC Custom Report Generator 2008 data [1] with adjustments of inflation rates for year 2015 according to Statistics Canada Consumer Price Index [2].

Table S3. Potential annual savings in healthcare and related costs of type 2 diabetes among Canadian adults from low glycaemic and/or high fiber diets that include pulses in Alberta (Can $ million).[1]

	**Scenario**			
	**Very pessimistic**	**Pessimistic**	**Optimistic**	**Very optimistic**
Direct cost savings				
Hospital care	0.1	0.2	0.3	0.6
Physician care	0.0	0.0	0.0	0.0
Drug	0.4	1.3	2.1	4.3
**Total direct cost savings**	**0.5**	**1.4**	**2.4**	**4.8**
Indirect cost savings[2]				
Due to Mortality	<0.1	<0.1	<0.1	<0.1
Due to Morbidity	NA	NA	NA	NA
**Total indirect cost savings**	**<0.1**	**<0.1**	**<0.1**	**<0.1**
**Total cost savings**	**0.5**	**1.5**	**2.4**	**4.9**

[1] Data represent type 2 diabetes-related financial savings following reduction in HbA1c concentrations with adoption of a low GI or high fiber diet that includes pulses for men and women [3]. Given that patients with T2D are expected to continue to visit their physicians, these costs remained unchanged. The very optimistic scenario is a long-term estimate of potential savings when 50% of Canadian adults (≥18 years of age) with T2D consume a low GI or high fiber diet with dietary pulses. The optimistic scenario is a medium-to-long-term pragmatic estimate of potential savings when 25% of adults in Canada with T2D use pulses to adopt a low GI or high fiber diet. The pessimistic and very pessimistic scenario is a practical short-to-medium-term, and immediate estimate of cost savings that could follow when 15% and 5% of adults with T2D follow a low GI or high fiber diet with pulses. NA, not available. [2] Indirect costs only include values of lost production due to reduced working time associated with illness, injury, or premature death, and do not include any valuation of morbidity and mortality themselves.

Table S4. Potential annual savings in healthcare and related costs of type 2 diabetes among Canadian adults from low glycaemic and/or high fiber diets that include pulses in British Columbia (Can $ million).[1]

	**Scenario**			
	**Very pessimistic**	**Pessimistic**	**Optimistic**	**Very optimistic**
Direct cost savings				
Hospital care	0.1	0.3	0.5	1.0
Physician care	0.0	0.0	0.0	0.0
Drug	0.4	1.2	2.1	4.1
**Total direct cost savings**	**0.5**	**1.5**	**2.5**	**5.1**
Indirect cost savings[2]				
Due to Mortality	<0.1	<0.1	<0.1	<0.1
Due to Morbidity	NA	NA	NA	NA
**Total indirect cost savings**	**<0.1**	**<0.1**	**<0.1**	**<0.1**
**Total cost savings**	**0.5**	**1.5**	**2.6**	**5.1**

[1] Data represent type 2 diabetes-related financial savings following reduction in HbA1c concentrations with adoption of a low GI or high fiber diet that includes pulses for men and women [3]. Given that patients with T2D are expected to continue to visit their physicians, these costs remained unchanged. The very optimistic scenario is a long-term estimate of potential savings when 50% of Canadian adults (≥18 years of age) with T2D consume a low GI or high fiber diet with dietary pulses. The optimistic scenario is a medium-to-long-term pragmatic estimate of potential savings when 25% of adults in Canada with T2D use pulses to adopt a low GI or high fiber diet. The pessimistic and very pessimistic scenario is a practical short-to-medium-term, and immediate estimate of cost savings that could follow when 15% and 5% of adults with T2D follow a low GI or high fiber diet with pulses. NA, not available. [2] Indirect costs only include values of lost production due to reduced working time associated with illness, injury, or premature death, and do not include any valuation of morbidity and mortality themselves.

Table S5. Potential annual savings in healthcare and related costs of type 2 diabetes among Canadian adults from low glycaemic and/or high fiber diets that include pulses in Manitoba (Can $ million).[1]

	**Scenario**			
	**Very pessimistic**	**Pessimistic**	**Optimistic**	**Very optimistic**
Direct cost savings				
Hospital care	<0.1	0.1	0.1	0.2
Physician care	0.0	0.0	0.0	0.0
Drug	0.1	0.4	0.7	1.4
**Total direct cost savings**	**0.2**	**0.5**	**0.8**	**1.6**
Indirect cost savings[2]				
Due to Mortality	<0.1	<0.1	<0.1	<0.1
Due to Morbidity	NA	NA	NA	NA
**Total indirect cost savings**	**<0.1**	**<0.1**	**<0.1**	**<0.1**
**Total cost savings**	**0.2**	**0.5**	**0.8**	**1.7**

[1] Data represent type 2 diabetes-related financial savings following reduction in HbA1c concentrations with adoption of a low GI or high fiber diet that includes pulses for men and women [3]. Given that patients with T2D are expected to continue to visit their physicians, these costs remained unchanged. The very optimistic scenario is a long-term estimate of potential savings when 50% of Canadian adults (≥18 years of age) with T2D consume a low GI or high fiber diet with dietary pulses. The optimistic scenario is a medium-to-long-term pragmatic estimate of potential savings when 25% of adults in Canada with T2D use pulses to adopt a low GI or high fiber diet. The pessimistic and very pessimistic scenario is a practical short-to-medium-term, and immediate estimate of cost savings that could follow when 15% and 5% of adults with T2D follow a low GI or high fiber diet with pulses. NA, not available. [2] Indirect costs only include values of lost production due to reduced working time associated with illness, injury, or premature death, and do not include any valuation of morbidity and mortality themselves.

Table S6. Potential annual savings in healthcare and related costs of type 2 diabetes among Canadian adults from low glycaemic and/or high fiber diets that include pulses in New Brunswick (Can $ million).[1]

	**Scenario**			
	**Very pessimistic**	**Pessimistic**	**Optimistic**	**Very optimistic**
Direct cost savings				
Hospital care	<0.1	0.1	0.1	0.2
Physician care	0.0	0.0	0.0	0.0
Drug	0.1	0.3	0.6	1.2
**Total direct cost savings**	**0.1**	**0.4**	**0.7**	**1.3**
Indirect cost savings[2]				
Due to Mortality	<0.1	<0.1	<0.1	<0.1
Due to Morbidity	NA	NA	NA	NA
**Total indirect cost savings**	**<0.1**	**<0.1**	**<0.1**	**<0.1**
**Total cost savings**	**0.1**	**0.4**	**0.7**	**1.3**

[1] Data represent type 2 diabetes-related financial savings following reduction in HbA1c concentrations with adoption of a low GI or high fiber diet that includes pulses for men and women [3]. Given that patients with T2D are expected to continue to visit their physicians, these costs remained unchanged. The very optimistic scenario is a long-term estimate of potential savings when 50% of Canadian adults (≥18 years of age) with T2D consume a low GI or high fiber diet with dietary pulses. The optimistic scenario is a medium-to-long-term pragmatic estimate of potential savings when 25% of adults in Canada with T2D use pulses to adopt a low GI or high fiber diet. The pessimistic and very pessimistic scenario is a practical short-to-medium-term, and immediate estimate of cost savings that could follow when 15% and 5% of adults with T2D follow a low GI or high fiber diet with pulses. NA, not available. [2] Indirect costs only include values of lost production due to reduced working time associated with illness, injury, or premature death, and do not include any valuation of morbidity and mortality themselves.

Table S7. Potential annual savings in healthcare and related costs of type 2 diabetes among Canadian adults from low glycaemic and/or high fiber diets that include pulses in Newfoundland and Labrador (Can $ million).[1]

	**Scenario**			
	**Very pessimistic**	**Pessimistic**	**Optimistic**	**Very optimistic**
Direct cost savings				
Hospital care	<0.1	<0.1	0.1	0.1
Physician care	0.0	0.0	0.0	0.0
Drug	0.1	0.3	0.4	0.9
**Total direct cost savings**	**0.1**	**0.3**	**0.5**	**1.0**
Indirect cost savings[2]				
Due to Mortality	<0.1	<0.1	<0.1	<0.1
Due to Morbidity	NA	NA	NA	NA
**Total indirect cost savings**	**<0.1**	**<0.1**	**<0.1**	**<0.1**
**Total cost savings**	**0.1**	**0.3**	**0.5**	**1.0**

[1] Data represent type 2 diabetes-related financial savings following reduction in HbA1c concentrations with adoption of a low GI or high fiber diet that includes pulses for men and women [3]. Given that patients with T2D are expected to continue to visit their physicians, these costs remained unchanged. The very optimistic scenario is a long-term estimate of potential savings when 50% of Canadian adults (≥18 years of age) with T2D consume a low GI or high fiber diet with dietary pulses. The optimistic scenario is a medium-to-long-term pragmatic estimate of potential savings when 25% of adults in Canada with T2D use pulses to adopt a low GI or high fiber diet. The pessimistic and very pessimistic scenario is a practical short-to-medium-term, and immediate estimate of cost savings that could follow when 15% and 5% of adults with T2D follow a low GI or high fiber diet with pulses. NA, not available. [2] Indirect costs only include values of lost production due to reduced working time associated with illness, injury, or premature death, and do not include any valuation of morbidity and mortality themselves.

Table S8. Potential annual savings in healthcare and related costs of type 2 diabetes among Canadian adults from low glycaemic and/or high fiber diets that include pulses in The Northwest Territories (Can $ million).[1]

	**Scenario**			
	**Very pessimistic**	**Pessimistic**	**Optimistic**	**Very optimistic**
Direct cost savings				
Hospital care	<0.1	<0.1	<0.1	<0.1
Physician care	0.0	0.0	0.0	0.0
Drug	<0.1	<0.1	<0.1	<0.1
**Total direct cost savings**	**<0.1**	**<0.1**	**<0.1**	**<0.1**
Indirect cost savings[2]				
Due to Mortality	<0.1	<0.1	<0.1	<0.1
Due to Morbidity	NA	NA	NA	NA
**Total indirect cost savings**	**<0.1**	**<0.1**	**<0.1**	**<0.1**
**Total cost savings**	**<0.1**	**<0.1**	**<0.1**	**<0.1**

[1] Data represent type 2 diabetes-related financial savings following reduction in HbA1c concentrations with adoption of a low GI or high fiber diet that includes pulses for men and women [3]. Given that patients with T2D are expected to continue to visit their physicians, these costs remained unchanged. The very optimistic scenario is a long-term estimate of potential savings when 50% of Canadian adults (≥18 years of age) with T2D consume a low GI or high fiber diet with dietary pulses. The optimistic scenario is a medium-to-long-term pragmatic estimate of potential savings when 25% of adults in Canada with T2D use pulses to adopt a low GI or high fiber diet. The pessimistic and very pessimistic scenario is a practical short-to-medium-term, and immediate estimate of cost savings that could follow when 15% and 5% of adults with T2D follow a low GI or high fiber diet with pulses. NA, not available. [2] Indirect costs only include values of lost production due to reduced working time associated with illness, injury, or premature death, and do not include any valuation of morbidity and mortality themselves.

Table S9. Potential annual savings in healthcare and related costs of type 2 diabetes among Canadian adults from low glycaemic and/or high fiber diets that include pulses in Nova Scotia (Can $ million).[1]

	**Scenario**			
	**Very pessimistic**	**Pessimistic**	**Optimistic**	**Very optimistic**
Direct cost savings				
Hospital care	<0.1	0.1	0.1	0.2
Physician care	0.0	0.0	0.0	0.0
Drug	0.1	0.4	0.7	1.5
**Total direct cost savings**	**0.2**	**0.5**	**0.8**	**1.7**
Indirect cost savings[2]				
Due to Mortality	<0.1	<0.1	<0.1	<0.1
Due to Morbidity	NA	NA	NA	NA
**Total indirect cost savings**	**<0.1**	**<0.1**	**<0.1**	**<0.1**
**Total cost savings**	**0.2**	**0.5**	**0.8**	**1.7**

[1] Data represent type 2 diabetes-related financial savings following reduction in HbA1c concentrations with adoption of a low GI or high fiber diet that includes pulses for men and women [3]. Given that patients with T2D are expected to continue to visit their physicians, these costs remained unchanged. The very optimistic scenario is a long-term estimate of potential savings when 50% of Canadian adults (≥18 years of age) with T2D consume a low GI or high fiber diet with dietary pulses. The optimistic scenario is a medium-to-long-term pragmatic estimate of potential savings when 25% of adults in Canada with T2D use pulses to adopt a low GI or high fiber diet. The pessimistic and very pessimistic scenario is a practical short-to-medium-term, and immediate estimate of cost savings that could follow when 15% and 5% of adults with T2D follow a low GI or high fiber diet with pulses. NA, not available. [2] Indirect costs only include values of lost production due to reduced working time associated with illness, injury, or premature death, and do not include any valuation of morbidity and mortality themselves.

Table S10. Potential annual savings in healthcare and related costs of type 2 diabetes among Canadian adults from low glycaemic and/or high fiber diets that include pulses in Nunavut (Can $ million).[1]

	**Scenario**			
	**Very pessimistic**	**Pessimistic**	**Optimistic**	**Very optimistic**
Direct cost savings				
Hospital care	<0.1	<0.1	<0.1	<0.1
Physician care	0.0	0.0	0.0	0.0
Drug	<0.1	<0.1	<0.1	<0.1
**Total direct cost savings**	**<0.1**	**<0.1**	**<0.1**	**<0.1**
Indirect cost savings[2]				
Due to Mortality	<0.1	<0.1	<0.1	<0.1
Due to Morbidity	NA	NA	NA	NA
**Total indirect cost savings**	**<0.1**	**<0.1**	**<0.1**	**<0.1**
**Total cost savings**	**<0.1**	**<0.1**	**<0.1**	**<0.1**

[1] Data represent type 2 diabetes-related financial savings following reduction in HbA1c concentrations with adoption of a low GI or high fiber diet that includes pulses for men and women [3]. Given that patients with T2D are expected to continue to visit their physicians, these costs remained unchanged. The very optimistic scenario is a long-term estimate of potential savings when 50% of Canadian adults (≥18 years of age) with T2D consume a low GI or high fiber diet with dietary pulses. The optimistic scenario is a medium-to-long-term pragmatic estimate of potential savings when 25% of adults in Canada with T2D use pulses to adopt a low GI or high fiber diet. The pessimistic and very pessimistic scenario is a practical short-to-medium-term, and immediate estimate of cost savings that could follow when 15% and 5% of adults with T2D follow a low GI or high fiber diet with pulses. NA, not available. [2] Indirect costs only include values of lost production due to reduced working time associated with illness, injury, or premature death, and do not include any valuation of morbidity and mortality themselves.

Table S11. Potential annual savings in healthcare and related costs of type 2 diabetes among Canadian adults from low glycaemic and/or high fiber diets that include pulses in Ontario (Can $ million).[1]

	Scenario			
	**Very pessimistic**	**Pessimistic**	**Optimistic**	**Very optimistic**
Direct cost savings				
Hospital care	0.2	0.6	0.9	1.9
Physician care	0.0	0.0	0.0	0.0
Drug	2.2	6.5	10.9	21.8
**Total direct cost savings**	**2.4**	**7.1**	**11.8**	**23.7**
Indirect cost savings[2]				
Due to Mortality	<0.1	0.1	0.1	0.3
Due to Morbidity	NA	NA	NA	NA
**Total indirect cost savings**	**<0.1**	**0.1**	**0.1**	**0.3**
**Total cost savings**	**2.4**	**7.2**	**12.0**	**23.9**

[1] Data represent type 2 diabetes-related financial savings following reduction in HbA1c concentrations with adoption of a low GI or high fiber diet that includes pulses for men and women [3]. Given that patients with T2D are expected to continue to visit their physicians, these costs remained unchanged. The very optimistic scenario is a long-term estimate of potential savings when 50% of Canadian adults (≥18 years of age) with T2D consume a low GI or high fiber diet with dietary pulses. The optimistic scenario is a medium-to-long-term pragmatic estimate of potential savings when 25% of adults in Canada with T2D use pulses to adopt a low GI or high fiber diet. The pessimistic and very pessimistic scenario is a practical short-to-medium-term, and immediate estimate of cost savings that could follow when 15% and 5% of adults with T2D follow a low GI or high fiber diet with pulses. NA, not available. [2] Indirect costs only include values of lost production due to reduced working time associated with illness, injury, or premature death, and do not include any valuation of morbidity and mortality themselves.

Table S12. Potential annual savings in healthcare and related costs of type 2 diabetes among Canadian adults from low glycaemic and/or high fiber diets that include pulses in Prince Edward Island (Can $ million).[1]

	**Scenario**			
	**Very pessimistic**	**Pessimistic**	**Optimistic**	**Very optimistic**
Direct cost savings				
Hospital care	<0.1	<0.1	<0.1	<0.1
Physician care	0.0	0.0	0.0	0.0
Drug	<0.1	0.1	0.1	0.2
**Total direct cost savings**	**<0.1**	**0.1**	**0.1**	**0.2**
Indirect cost savings[2]				
Due to Mortality	<0.1	<0.1	<0.1	<0.1
Due to Morbidity	NA	NA	NA	NA
**Total indirect cost savings**	**<0.1**	**<0.1**	**<0.1**	**<0.1**
**Total cost savings**	**<0.1**	**0.1**	**0.1**	**0.2**

[1] Data represent type 2 diabetes-related financial savings following reduction in HbA1c concentrations with adoption of a low GI or high fiber diet that includes pulses for men and women [3]. Given that patients with T2D are expected to continue to visit their physicians, these costs remained unchanged. The very optimistic scenario is a long-term estimate of potential savings when 50% of Canadian adults (≥18 years of age) with T2D consume a low GI or high fiber diet with dietary pulses. The optimistic scenario is a medium-to-long-term pragmatic estimate of potential savings when 25% of adults in Canada with T2D use pulses to adopt a low GI or high fiber diet. The pessimistic and very pessimistic scenario is a practical short-to-medium-term, and immediate estimate of cost savings that could follow when 15% and 5% of adults with T2D follow a low GI or high fiber diet with pulses. NA, not available. [2] Indirect costs only include values of lost production due to reduced working time associated with illness, injury, or premature death, and do not include any valuation of morbidity and mortality themselves.

Table S13. Potential annual savings in healthcare and related costs of type 2 diabetes among Canadian adults from low glycaemic and/or high fiber diets that include pulses in Quebec (Can $ million).[1]

	**Scenario**			
	**Very pessimistic**	**Pessimistic**	**Optimistic**	**Very optimistic**
Direct cost savings				
Hospital care	0.1	0.2	0.3	0.7
Physician care	0.0	0.0	0.0	0.0
Drug	1.2	3.5	5.8	11.6
**Total direct cost savings**	**1.2**	**3.7**	**6.1**	**12.2**
Indirect cost savings[2]				
Due to Mortality	<0.1	<0.1	<0.1	0.1
Due to Morbidity	NA	NA	NA	NA
**Total indirect cost savings**	**<0.1**	**<0.1**	**<0.1**	**0.1**
**Total cost savings**	**1.2**	**3.7**	**6.2**	**12.3**

[1] Data represent type 2 diabetes-related financial savings following reduction in HbA1c concentrations with adoption of a low GI or high fiber diet that includes pulses for men and women [3]. Given that patients with T2D are expected to continue to visit their physicians, these costs remained unchanged. The very optimistic scenario is a long-term estimate of potential savings when 50% of Canadian adults (≥18 years of age) with T2D consume a low GI or high fiber diet with dietary pulses. The optimistic scenario is a medium-to-long-term pragmatic estimate of potential savings when 25% of adults in Canada with T2D use pulses to adopt a low GI or high fiber diet. The pessimistic and very pessimistic scenario is a practical short-to-medium-term, and immediate estimate of cost savings that could follow when 15% and 5% of adults with T2D follow a low GI or high fiber diet with pulses. NA, not available. [2] Indirect costs only include values of lost production due to reduced working time associated with illness, injury, or premature death, and do not include any valuation of morbidity and mortality themselves.

Table S14. Potential annual savings in healthcare and related costs of type 2 diabetes among Canadian adults from low glycaemic and/or high fiber diets that include pulses in Saskatchewan (Can $ million).[1]

	**Scenario**			
	**Very pessimistic**	**Pessimistic**	**Optimistic**	**Very optimistic**
Direct cost savings				
Hospital care	<0.1	<0.1	0.1	0.2
Physician care	0.0	0.0	0.0	0.0
Drug	0.1	0.4	0.6	1.2
**Total direct cost savings**	**0.1**	**0.4**	**0.7**	**1.4**
Indirect cost savings[2]				
Due to Mortality	<0.1	<0.1	<0.1	<0.1
Due to Morbidity	NA	NA	NA	NA
**Total indirect cost savings**	**<0.1**	**<0.1**	**<0.1**	**<0.1**
**Total cost savings**	**0.1**	**0.4**	**0.7**	**1.4**

[1] Data represent type 2 diabetes-related financial savings following reduction in HbA1c concentrations with adoption of a low GI or high fiber diet that includes pulses for men and women [3]. Given that patients with T2D are expected to continue to visit their physicians, these costs remained unchanged. The very optimistic scenario is a long-term estimate of potential savings when 50% of Canadian adults (≥18 years of age) with T2D consume a low GI or high fiber diet with dietary pulses. The optimistic scenario is a medium-to-long-term pragmatic estimate of potential savings when 25% of adults in Canada with T2D use pulses to adopt a low GI or high fiber diet. The pessimistic and very pessimistic scenario is a practical short-to-medium-term, and immediate estimate of cost savings that could follow when 15% and 5% of adults with T2D follow a low GI or high fiber diet with pulses. NA, not available. [2] Indirect costs only include values of lost production due to reduced working time associated with illness, injury, or premature death, and do not include any valuation of morbidity and mortality themselves.

Table S15. Potential annual savings in healthcare and related costs of type 2 diabetes among Canadian adults from low glycaemic and/or high fiber diets that include pulses in Yukon (Can $ million).[1]

	**Scenario**			
	**Very pessimistic**	**Pessimistic**	**Optimistic**	**Very optimistic**
Direct cost savings				
Hospital care	<0.1	<0.1	<0.1	<0.1
Physician care	0.0	0.0	0.0	0.0
Drug	<0.1	<0.1	<0.1	<0.1
**Total direct cost savings**	**<0.1**	**<0.1**	**<0.1**	**<0.1**
Indirect cost savings[2]				
Due to Mortality	<0.1	<0.1	<0.1	<0.1
Due to Morbidity	NA	NA	NA	NA
**Total indirect cost savings**	**<0.1**	**<0.1**	**<0.1**	**<0.1**
**Total cost savings**	**<0.1**	**<0.1**	**<0.1**	**<0.1**

[1] Data represent type 2 diabetes-related financial savings following reduction in HbA1c concentrations with adoption of a low GI or high fiber diet that includes pulses for men and women [3]. Given that patients with T2D are expected to continue to visit their physicians, these costs remained unchanged. The very optimistic scenario is a long-term estimate of potential savings when 50% of Canadian adults (≥18 years of age) with T2D consume a low GI or high fiber diet with dietary pulses. The optimistic scenario is a medium-to-long-term pragmatic estimate of potential savings when 25% of adults in Canada with T2D use pulses to adopt a low GI or high fiber diet. The pessimistic and very pessimistic scenario is a practical short-to-medium-term, and immediate estimate of cost savings that could follow when 15% and 5% of adults with T2D follow a low GI or high fiber diet with pulses. NA, not available. [2] Indirect costs only include values of lost production due to reduced working time associated with illness, injury, or premature death, and do not include any valuation of morbidity and mortality themselves.

Table S16. Potential annual savings in healthcare and related costs of cardiovascular disease among Canadian adults from 100 g/day dietary pulse intake in Alberta (Can $ million).[1]

	Scenario			
	**Very pessimistic**	**Pessimistic**	**Optimistic**	**Very optimistic**
**Cost savings following LDL-C reduction**				
Direct cost savings				
Hospital care	0.2	0.7	1.2	2.3
Physician care	0.6	1.7	2.8	5.6
Drug	0.8	2.3	3.8	7.6
**Total direct cost savings**	**1.6**	**4.7**	**7.8**	**15.5**
Indirect cost savings[2]				
Due to Mortality	<0.1	0.1	0.1	0.2
Due to Morbidity	NA	NA	NA	NA
**Total indirect cost savings**	**<0.1**	**0.1**	**0.1**	**0.2**
**Total cost savings**	**1.6**	**4.7**	**7.9**	**15.7**
**Cost savings following SBP reduction**				
Direct cost savings				
Hospital care	0.2	0.5	0.9	1.8
Physician care	0.4	1.3	2.2	4.5
Drug	0.6	1.8	3.0	6.1
**Total direct cost savings**	**1.2**	**3.7**	**6.2**	**12.4**
Indirect cost savings[2]				
Due to Mortality	<0.1	<0.1	0.1	0.1
Due to Morbidity	NA	NA	NA	NA
**Total indirect cost savings**	**<0.1**	**<0.1**	**0.1**	**0.1**
**Total cost savings**	**1.2**	**3.7**	**6.2**	**12.5**
**Cost savings following LDL-C and SBP reduction combined**				
Direct cost savings				
Hospital care	0.4	1.2	2.1	4.1
Physician care	1.0	3.0	5.0	10.1
Drug	1.4	4.1	6.8	13.7
**Total direct cost savings**	**2.8**	**8.4**	**14**	**27.9**
Indirect cost savings[2]				
Due to Mortality	<0.1	<0.1	0.2	0.3
Due to Morbidity	NA	NA	NA	NA
**Total indirect cost savings**	**<0.1**	**<0.1**	**0.2**	**0.3**
**Total cost savings**	**2.8**	**8.4**	**14.1**	**28.2**

[1] Data represent cardiovascular disease-related financial savings following reductions in LDL-cholesterol concentrations and systolic blood pressure with the consumption of 100 g/day pulses for men and women [3]. The very optimistic scenario is an estimate of potential savings when 50% of Canadian adults (≥18 years of age)

consume one 100 g/day serving of dietary pulses. The optimistic scenario is a medium-to-long-term pragmatic estimate of potential savings when 25% of adults in Canada consume pulse regularly. The pessimistic scenario is a practical short-to-medium-term estimate of cost savings that could follow the dietary pulse consumptions among 15% of adults. The very pessimistic scenario is an immediate estimate when 5% of Canadian adults adopt 100 g/day serving of pulses. LDL-C, LDL-cholesterol; NA, not available; SBP, systolic blood pressure. [2] Indirect costs only include values of lost production due to reduced working time associated with illness, injury, or premature death, and do not include any valuation of morbidity and mortality themselves.

Table S17. Potential annual savings in healthcare and related costs of cardiovascular disease among Canadian adults from 100 g/day dietary pulse intake in British Columbia (Can $ million).[1]

	Scenario			
	**Very pessimistic**	**Pessimistic**	**Optimistic**	**Very optimistic**
**Cost savings following LDL-C reduction**				
Direct cost savings				
Hospital care	0.4	1.2	1.9	3.8
Physician care	0.7	2.0	3.3	6.6
Drug	0.9	2.6	4.3	8.6
**Total direct cost savings**	**1.9**	**5.7**	**9.5**	**19.1**
Indirect cost savings[2]				
Due to Mortality	<0.1	0.1	0.1	0.2
Due to Morbidity	NA	NA	NA	NA
**Total indirect cost savings**	**<0.1**	**0.1**	**0.1**	**0.2**
**Total cost savings**	**1.9**	**5.8**	**9.6**	**19.3**
**Cost savings following SBP reduction**				
Direct cost savings				
Hospital care	0.3	0.9	1.5	3.1
Physician care	0.5	1.6	2.6	5.2
Drug	0.7	2.1	3.4	6.9
**Total direct cost savings**	**1.5**	**4.5**	**7.6**	**15.1**
Indirect cost savings[2]				
Due to Mortality	<0.1	<0.1	0.1	0.2
Due to Morbidity	<0.1	<0.1	<0.1	<0.1
**Total indirect cost savings**	**<0.1**	**<0.1**	**0.1**	**0.2**
**Total cost savings**	**1.5**	**4.6**	**7.7**	**15.3**
**Cost savings following LDL-C and SBP reduction combined**				
Direct cost savings				
Hospital care	0.7	2.1	3.4	6.9
Physician care	1.2	3.6	5.9	11.8
Drug	1.6	4.7	7.7	15.5
**Total direct cost savings**	**3.4**	**10.2**	**17.1**	**34.2**
Indirect cost savings[2]				
Due to Mortality	<0.1	<0.1	0.2	0.4
Due to Morbidity	NA	NA	NA	NA
**Total indirect cost savings**	**<0.1**	**<0.1**	**0.2**	**0.4**
**Total cost savings**	**3.4**	**10.4**	**17.3**	**34.6**

[1] Data represent cardiovascular disease-related financial savings following reductions in LDL-cholesterol concentrations and systolic blood pressure with the consumption of 100 g/day pulses for men and women [3]. The very optimistic scenario is an estimate of potential savings when 50% of Canadian adults (≥18 years of age)

consume one 100 g/day serving of dietary pulses. The optimistic scenario is a medium-to-long-term pragmatic estimate of potential savings when 25% of adults in Canada consume pulse regularly. The pessimistic scenario is a practical short-to-medium-term estimate of cost savings that could follow the dietary pulse consumptions among 15% of adults. The very pessimistic scenario is an immediate estimate when 5% of Canadian adults adopt 100 g/day serving of pulses. LDL-C, LDL-cholesterol; NA, not available; SBP, systolic blood pressure. [2] Indirect costs only include values of lost production due to reduced working time associated with illness, injury, or premature death, and do not include any valuation of morbidity and mortality themselves.

Table S18. Potential annual savings in healthcare and related costs of cardiovascular disease among Canadian adults from 100 g/day dietary pulse intake in Manitoba (Can $ million).[1]

	Scenario			
	**Very pessimistic**	**Pessimistic**	**Optimistic**	**Very optimistic**
**Cost savings following LDL-C reduction**				
Direct cost savings				
Hospital care	0.1	0.3	0.5	1.0
Physician care	0.2	0.5	0.8	1.7
Drug	0.3	0.9	1.6	3.1
**Total direct cost savings**	**0.6**	**1.7**	**2.9**	**5.8**
Indirect cost savings[2]				
Due to Mortality	<0.1	<0.1	<0.1	0.1
Due to Morbidity	NA	NA	NA	NA
**Total indirect cost savings**	**<0.1**	**<0.1**	**<0.1**	**0.1**
**Total cost savings**	**0.6**	**1.8**	**2.9**	**5.9**
**Cost savings following SBP reduction**				
Direct cost savings				
Hospital care	0.1	0.2	0.4	0.8
Physician care	0.1	0.4	0.7	1.3
Drug	0.2	0.7	1.2	2.5
**Total direct cost savings**	**0.5**	**1.4**	**2.3**	**4.6**
Indirect cost savings[2]				
Due to Mortality	<0.1	<0.1	<0.1	0.1
Due to Morbidity	NA	NA	NA	NA
**Total indirect cost savings**	**<0.1**	**<0.1**	**<0.1**	**0.1**
**Total cost savings**	**0.5**	**1.4**	**2.3**	**4.7**
**Cost savings following LDL-C and SBP reduction combined**				
Direct cost savings				
Hospital care	0.2	0.5	0.9	1.8
Physician care	0.3	0.9	1.5	3.0
Drug	0.5	1.6	2.8	5.6
**Total direct cost savings**	**1.1**	**3.1**	**5.2**	**10.4**
Indirect cost savings[2]				
Due to Mortality	<0.1	<0.1	<0.1	0.2
Due to Morbidity	NA	NA	NA	NA
**Total indirect cost savings**	**<0.1**	**<0.1**	**<0.1**	**0.2**
**Total cost savings**	**1.1**	**3.2**	**5.2**	**10.6**

[1] Data represent cardiovascular disease-related financial savings following reductions in LDL-cholesterol concentrations and systolic blood pressure with the consumption of 100 g/day pulses for men and women [3]. The very optimistic scenario is an estimate of potential savings when 50% of Canadian adults (≥18 years of age)

consume one 100 g/day serving of dietary pulses. The optimistic scenario is a medium-to-long-term pragmatic estimate of potential savings when 25% of adults in Canada consume pulse regularly. The pessimistic scenario is a practical short-to-medium-term estimate of cost savings that could follow the dietary pulse consumptions among 15% of adults. The very pessimistic scenario is an immediate estimate when 5% of Canadian adults adopt 100 g/day serving of pulses. LDL-C, LDL-cholesterol; NA, not available; SBP, systolic blood pressure. [2] Indirect costs only include values of lost production due to reduced working time associated with illness, injury, or premature death, and do not include any valuation of morbidity and mortality themselves.

Table S19. Potential annual savings in healthcare and related costs of cardiovascular disease among Canadian adults from 100 g/day dietary pulse intake in New Brunswick (Can $ million).[1]

	Scenario			
	Very pessimistic	Pessimistic	Optimistic	Very optimistic
**Cost savings following LDL-C reduction**				
Direct cost savings				
Hospital care	0.1	0.2	0.3	0.7
Physician care	0.1	0.3	0.5	1.0
Drug	0.2	0.6	1.1	2.2
**Total direct cost savings**	**0.4**	**1.1**	**1.9**	**3.8**
Indirect cost savings[2]				
Due to Mortality	<0.1	<0.1	<0.1	<0.1
Due to Morbidity	NA	NA	NA	NA
**Total indirect cost savings**	**<0.1**	**<0.1**	**<0.1**	**<0.1**
**Total cost savings**	**0.4**	**1.2**	**1.9**	**3.9**
**Cost savings following SBP reduction**				
Direct cost savings				
Hospital care	0.1	0.2	0.3	0.5
Physician care	0.1	0.2	0.4	0.8
Drug	0.2	0.5	0.9	1.7
**Total direct cost savings**	**0.3**	**0.9**	**1.5**	**3.0**
Indirect cost savings[2]				
Due to Mortality	<0.1	<0.1	<0.1	<0.1
Due to Morbidity	NA	NA	NA	NA
**Total indirect cost savings**	**<0.1**	**<0.1**	**<0.1**	**<0.1**
**Total cost savings**	**0.3**	**0.9**	**1.5**	**3.1**
**Cost savings following LDL-C and SBP reduction combined**				
Direct cost savings				
Hospital care	0.2	0.4	0.6	1.2
Physician care	0.2	0.5	0.9	1.8
Drug	0.4	1.1	2	3.9
**Total direct cost savings**	**0.7**	**2**	**3.4**	**6.8**
Indirect cost savings[2]				
Due to Mortality	<0.1	<0.1	<0.1	<0.1
Due to Morbidity	NA	NA	NA	NA
**Total indirect cost savings**	**<0.1**	**<0.1**	**<0.1**	**<0.1**
**Total cost savings**	**0.7**	**2.1**	**3.4**	**7.0**

[1] Data represent cardiovascular disease-related financial savings following reductions in LDL-cholesterol concentrations and systolic blood pressure with the consumption of 100 g/day pulses for men and women [3]. The very optimistic scenario is an estimate of potential savings when 50% of Canadian adults (≥18 years of age)

consume one 100 g/day serving of dietary pulses. The optimistic scenario is a medium-to-long-term pragmatic estimate of potential savings when 25% of adults in Canada consume pulse regularly. The pessimistic scenario is a practical short-to-medium-term estimate of cost savings that could follow the dietary pulse consumptions among 15% of adults. The very pessimistic scenario is an immediate estimate when 5% of Canadian adults adopt 100 g/day serving of pulses. LDL-C, LDL-cholesterol; NA, not available; SBP, systolic blood pressure. [2] Indirect costs only include values of lost production due to reduced working time associated with illness, injury, or premature death, and do not include any valuation of morbidity and mortality themselves.

Table S20. Potential annual savings in healthcare and related costs of cardiovascular disease among Canadian adults from 100 g/day dietary pulse intake in Newfoundland and Labrador (Can $ million).[1]

	Scenario			
	**Very pessimistic**	**Pessimistic**	**Optimistic**	**Very optimistic**
**Cost savings following LDL-C reduction**				
Direct cost savings				
Hospital care	0.1	0.2	0.3	0.6
Physician care	0.1	0.2	0.3	0.6
Drug	0.2	0.5	0.8	1.6
**Total direct cost savings**	**0.3**	**0.8**	**1.4**	**2.8**
Indirect cost savings[2]				
Due to Mortality	<0.1	<0.1	<0.1	<0.1
Due to Morbidity	NA	NA	NA	NA
**Total indirect cost savings**	**<0.1**	**<0.1**	**<0.1**	**<0.1**
**Total cost savings**	**0.3**	**0.9**	**1.4**	**2.8**
**Cost savings following SBP reduction**				
Direct cost savings				
Hospital care	0.0	0.1	0.2	0.5
Physician care	0.1	0.2	0.3	0.5
Drug	0.1	0.4	0.6	1.3
**Total direct cost savings**	**0.2**	**0.7**	**1.1**	**2.2**
Indirect cost savings[2]				
Due to Mortality	<0.1	<0.1	<0.1	<0.1
Due to Morbidity	NA	NA	NA	NA
**Total indirect cost savings**	**<0.1**	**<0.1**	**<0.1**	**<0.1**
**Total cost savings**	**0.2**	**0.7**	**1.1**	**2.3**
**Cost savings following LDL-C and SBP reduction combined**				
Direct cost savings				
Hospital care	0.1	0.3	0.5	1.1
Physician care	0.2	0.4	0.6	1.1
Drug	0.3	0.9	1.4	2.9
**Total direct cost savings**	**0.5**	**1.5**	**2.5**	**5.1**
Indirect cost savings[2]				
Due to Mortality	<0.1	<0.1	<0.1	<0.1
Due to Morbidity	NA	NA	NA	NA
**Total indirect cost savings**	**<0.1**	**<0.1**	**<0.1**	**<0.1**
**Total cost savings**	**0.5**	**1.6**	**2.5**	**5.1**

[1] Data represent cardiovascular disease-related financial savings following reductions in LDL-cholesterol concentrations and systolic blood pressure with the consumption of 100 g/day pulses for men and women [3].

The very optimistic scenario is an estimate of potential savings when 50% of Canadian adults (≥18 years of age) consume one 100 g/day serving of dietary pulses. The optimistic scenario is a medium-to-long-term pragmatic estimate of potential savings when 25% of adults in Canada consume pulse regularly. The pessimistic scenario is a practical short-to-medium-term estimate of cost savings that could follow the dietary pulse consumptions among 15% of adults. The very pessimistic scenario is an immediate estimate when 5% of Canadian adults adopt 100 g/day serving of pulses. LDL-C, LDL-cholesterol; NA, not available; SBP, systolic blood pressure. [2] Indirect costs only include values of lost production due to reduced working time associated with illness, injury, or premature death, and do not include any valuation of morbidity and mortality themselves.

Table S21. Potential annual savings in healthcare and related costs of cardiovascular disease among Canadian adults from 100 g/day dietary pulse intake in The Northwest Territories (Can $ million).[1]

	Scenario			
	Very pessimistic	Pessimistic	Optimistic	Very optimistic
**Cost savings following LDL-C reduction**				
Direct cost savings				
Hospital care	<0.1	<0.1	<0.1	<0.1
Physician care	<0.1	<0.1	<0.1	0.1
Drug	<0.1	<0.1	<0.1	<0.1
**Total direct cost savings**	**<0.1**	**<0.1**	**0.1**	**0.1**
Indirect cost savings[2]				
Due to Mortality	<0.1	<0.1	<0.1	<0.1
Due to Morbidity	NA	NA	NA	NA
**Total indirect cost savings**	**<0.1**	**<0.1**	**<0.1**	**<0.1**
**Total cost savings**	**<0.1**	**<0.1**	**0.1**	**0.1**
**Cost savings following SBP reduction**				
Direct cost savings				
Hospital care	<0.1	<0.1	<0.1	<0.1
Physician care	<0.1	<0.1	<0.1	0.1
Drug	<0.1	<0.1	<0.1	<0.1
**Total direct cost savings**	**<0.1**	**<0.1**	**0.1**	**0.1**
Indirect cost savings[2]				
Due to Mortality	<0.1	<0.1	<0.1	<0.1
Due to Morbidity	NA	NA	NA	NA
**Total indirect cost savings**	**<0.1**	**<0.1**	**<0.1**	**<0.1**
**Total cost savings**	**<0.1**	**<0.1**	**0.1**	**0.1**
**Cost savings following LDL-C and SBP reduction combined**				
Direct cost savings				
Hospital care	<0.1	<0.1	<0.1	<0.1
Physician care	<0.1	<0.1	<0.1	0.2
Drug	<0.1	<0.1	<0.1	<0.1
**Total direct cost savings**	**<0.1**	**<0.1**	**0.2**	**0.2**
Indirect cost savings[2]				
Due to Mortality	<0.1	<0.1	<0.1	<0.1
Due to Morbidity	NA	NA	NA	NA
**Total indirect cost savings**	**<0.1**	**<0.1**	**<0.1**	**<0.1**
**Total cost savings**	**<0.1**	**<0.1**	**0.2**	**0.2**

[1] Data represent cardiovascular disease-related financial savings following reductions in LDL-cholesterol concentrations and systolic blood pressure with the consumption of 100 g/day pulses for men and women [3]. The very optimistic scenario is an estimate of potential savings when 50% of Canadian adults (≥18 years of age)

consume one 100 g/day serving of dietary pulses. The optimistic scenario is a medium-to-long-term pragmatic estimate of potential savings when 25% of adults in Canada consume pulse regularly. The pessimistic scenario is a practical short-to-medium-term estimate of cost savings that could follow the dietary pulse consumptions among 15% of adults. The very pessimistic scenario is an immediate estimate when 5% of Canadian adults adopt 100 g/day serving of pulses. LDL-C, LDL-cholesterol; NA, not available; SBP, systolic blood pressure. [2] Indirect costs only include values of lost production due to reduced working time associated with illness, injury, or premature death, and do not include any valuation of morbidity and mortality themselves.

Table S22. Potential annual savings in healthcare and related costs of cardiovascular disease among Canadian adults from 100 g/day dietary pulse intake in Nova Scotia (Can $ million).[1]

	Scenario			
	Very pessimistic	Pessimistic	Optimistic	Very optimistic
**Cost savings following LDL-C reduction**				
Direct cost savings				
Hospital care	0.1	0.3	0.4	0.9
Physician care	0.1	0.4	0.6	1.2
Drug	0.3	0.8	1.4	2.8
**Total direct cost savings**	**0.5**	**1.5**	**2.5**	**4.9**
Indirect cost savings[2]				
Due to Mortality	<0.1	<0.1	<0.1	0.1
Due to Morbidity	NA	NA	NA	NA
**Total indirect cost savings**	**<0.1**	**<0.1**	**<0.1**	**0.1**
**Total cost savings**	**0.5**	**1.5**	**2.5**	**5.0**
**Cost savings following SBP reduction**				
Direct cost savings				
Hospital care	0.1	0.2	0.4	0.7
Physician care	0.1	0.3	0.5	1.0
Drug	0.2	0.7	1.1	2.2
**Total direct cost savings**	**0.4**	**1.2**	**2.0**	**3.9**
Indirect cost savings[2]				
Due to Mortality	<0.1	<0.1	<0.1	<0.1
Due to Morbidity	NA	NA	NA	NA
**Total indirect cost savings**	**<0.1**	**<0.1**	**<0.1**	**<0.1**
**Total cost savings**	**0.4**	**1.2**	**2.0**	**3.9**
**Cost savings following LDL-C and SBP reduction combined**				
Direct cost savings				
Hospital care	0.2	0.5	0.8	1.6
Physician care	0.2	0.7	1.1	2.2
Drug	0.5	1.5	2.5	5.0
**Total direct cost savings**	**0.9**	**2.7**	**4.5**	**8.8**
Indirect cost savings[2]				
Due to Mortality	<0.1	<0.1	<0.1	<0.1
Due to Morbidity	NA	NA	NA	NA
**Total indirect cost savings**	**<0.1**	**<0.1**	**<0.1**	**<0.1**
**Total cost savings**	**0.9**	**2.7**	**4.5**	**8.9**

[1] Data represent cardiovascular disease-related financial savings following reductions in LDL-cholesterol concentrations and systolic blood pressure with the consumption of 100 g/day pulses for men and women [3]. The very optimistic scenario is an estimate of potential savings when 50% of Canadian adults (≥18 years of age)

consume one 100 g/day serving of dietary pulses. The optimistic scenario is a medium-to-long-term pragmatic estimate of potential savings when 25% of adults in Canada consume pulse regularly. The pessimistic scenario is a practical short-to-medium-term estimate of cost savings that could follow the dietary pulse consumptions among 15% of adults. The very pessimistic scenario is an immediate estimate when 5% of Canadian adults adopt 100 g/day serving of pulses. LDL-C, LDL-cholesterol; NA, not available; SBP, systolic blood pressure. [2] Indirect costs only include values of lost production due to reduced working time associated with illness, injury, or premature death, and do not include any valuation of morbidity and mortality themselves.

Table S23. Potential annual savings in healthcare and related costs of cardiovascular disease among Canadian adults from 100 g/day dietary pulse intake in Nunavut (Can $ million).[1]

	Scenario			
	**Very pessimistic**	**Pessimistic**	**Optimistic**	**Very optimistic**
**Cost savings following LDL-C reduction**				
Direct cost savings				
Hospital care	<0.1	<0.1	<0.1	<0.1
Physician care	<0.1	<0.1	<0.1	0.1
Drug	<0.1	<0.1	<0.1	<0.1
**Total direct cost savings**	**<0.1**	**<0.1**	**<0.1**	**0.1**
Indirect cost savings[2]				
Due to Mortality	<0.1	<0.1	<0.1	<0.1
Due to Morbidity	NA	NA	NA	NA
**Total indirect cost savings**	**<0.1**	**<0.1**	**<0.1**	**<0.1**
**Total cost savings**	**<0.1**	**<0.1**	**<0.1**	**0.1**
**Cost savings following SBP reduction**				
Direct cost savings				
Hospital care	<0.1	<0.1	<0.1	<0.1
Physician care	<0.1	<0.1	<0.1	0.1
Drug	<0.1	<0.1	<0.1	<0.1
**Total direct cost savings**	**<0.1**	**<0.1**	**<0.1**	**0.1**
Indirect cost savings[2]				
Due to Mortality	<0.1	<0.1	<0.1	<0.1
Due to Morbidity	NA	NA	NA	NA
**Total indirect cost savings**	**<0.1**	**<0.1**	**<0.1**	**<0.1**
**Total cost savings**	**<0.1**	**<0.1**	**<0.1**	**0.1**
**Cost savings following LDL-C and SBP reduction combined**				
Direct cost savings				
Hospital care	<0.1	<0.1	<0.1	<0.1
Physician care	<0.1	<0.1	<0.1	0.2
Drug	<0.1	<0.1	<0.1	<0.1
**Total direct cost savings**	**<0.1**	**<0.1**	**<0.1**	**0.2**
Indirect cost savings[2]				
Due to Mortality	<0.1	<0.1	<0.1	<0.1
Due to Morbidity	NA	NA	NA	NA
**Total indirect cost savings**	**<0.1**	**<0.1**	**<0.1**	**<0.1**
**Total cost savings**	**<0.1**	**<0.1**	**<0.1**	**0.2**

[1] Data represent cardiovascular disease-related financial savings following reductions in LDL-cholesterol concentrations and systolic blood pressure with the consumption of 100 g/day pulses for men and women [3]. The very optimistic scenario is an estimate of potential savings when 50% of Canadian adults (≥18 years of age)

consume one 100 g/day serving of dietary pulses. The optimistic scenario is a medium-to-long-term pragmatic estimate of potential savings when 25% of adults in Canada consume pulse regularly. The pessimistic scenario is a practical short-to-medium-term estimate of cost savings that could follow the dietary pulse consumptions among 15% of adults. The very pessimistic scenario is an immediate estimate when 5% of Canadian adults adopt 100 g/day serving of pulses. LDL-C, LDL-cholesterol; NA, not available; SBP, systolic blood pressure. [2] Indirect costs only include values of lost production due to reduced working time associated with illness, injury, or premature death, and do not include any valuation of morbidity and mortality themselves.

Table S24. Potential annual savings in healthcare and related costs of cardiovascular disease among Canadian adults from 100 g/day dietary pulse intake in Ontario (Can $ million).[1]

	Scenario			
	**Very pessimistic**	**Pessimistic**	**Optimistic**	**Very optimistic**
**Cost savings following LDL-C reduction**				
Direct cost savings				
Hospital care	0.9	2.8	4.6	9.2
Physician care	2.0	6.1	10.2	20.4
Drug	3.2	9.5	15.8	31.6
**Total direct cost savings**	**6.1**	**18.4**	**30.6**	**61.2**
Indirect cost savings[2]				
Due to Mortality	0.1	0.2	0.4	0.8
Due to Morbidity	NA	NA	NA	NA
**Total indirect cost savings**	**0.1**	**0.2**	**0.4**	**0.8**
**Total cost savings**	**6.2**	**18.6**	**31.0**	**62.0**
**Cost savings following SBP reduction**				
Direct cost savings				
Hospital care	0.7	2.2	3.7	7.3
Physician care	1.6	4.9	8.1	16.2
Drug	2.5	7.5	12.5	25.1
**Total direct cost savings**	**4.9**	**14.6**	**24.3**	**48.6**
Indirect cost savings[2]				
Due to Mortality	0.1	0.2	0.3	0.6
Due to Morbidity	NA	NA	NA	NA
**Total indirect cost savings**	**0.1**	**0.2**	**0.3**	**0.6**
**Total cost savings**	**4.9**	**14.8**	**24.6**	**49.3**
**Cost savings following LDL-C and SBP reduction combined**				
Direct cost savings				
Hospital care	1.6	5.0	8.3	16.5
Physician care	3.6	11	18.3	36.6
Drug	5.7	17	28.3	56.7
**Total direct cost savings**	**11**	**33**	**54.9**	**109.8**
Indirect cost savings[2]				
Due to Mortality	0.2	0.4	0.7	1.4
Due to Morbidity	NA	NA	NA	NA
**Total indirect cost savings**	**0.2**	**0.4**	**0.7**	**1.4**
**Total cost savings**	**11.1**	**33.4**	**55.6**	**111.3**

[1] Data represent cardiovascular disease-related financial savings following reductions in LDL-cholesterol concentrations and systolic blood pressure with the consumption of 100 g/day pulses for men and women [3]. The very optimistic scenario is an estimate of potential savings when 50% of Canadian adults (≥18 years of age)

consume one 100 g/day serving of dietary pulses. The optimistic scenario is a medium-to-long-term pragmatic estimate of potential savings when 25% of adults in Canada consume pulse regularly. The pessimistic scenario is a practical short-to-medium-term estimate of cost savings that could follow the dietary pulse consumptions among 15% of adults. The very pessimistic scenario is an immediate estimate when 5% of Canadian adults adopt 100 g/day serving of pulses. LDL-C, LDL-cholesterol; NA, not available; SBP, systolic blood pressure. [2] Indirect costs only include values of lost production due to reduced working time associated with illness, injury, or premature death, and do not include any valuation of morbidity and mortality themselves.

Table S25. Potential annual savings in healthcare and related costs of cardiovascular disease among Canadian adults from 100 g/day dietary pulse intake in Prince Edward Island (Can $ million).[1]

	Scenario			
	**Very pessimistic**	**Pessimistic**	**Optimistic**	**Very optimistic**
**Cost savings following LDL-C reduction**				
Direct cost savings				
Hospital care	<0.1	<0.1	0.1	0.1
Physician care	<0.1	0.1	0.1	0.2
Drug	<0.1	0.1	0.2	0.4
**Total direct cost savings**	**0.1**	**0.2**	**0.3**	**0.7**
Indirect cost savings[2]				
Due to Mortality	<0.1	<0.1	<0.1	<0.1
Due to Morbidity	NA	NA	NA	NA
**Total indirect cost savings**	**<0.1**	**<0.1**	**<0.1**	**<0.1**
**Total cost savings**	**0.1**	**0.2**	**0.3**	**0.7**
**Cost savings following SBP reduction**				
Direct cost savings				
Hospital care	<0.1	<0.1	<0.1	0.1
Physician care	<0.1	<0.1	0.1	0.1
Drug	<0.1	0.1	0.2	0.3
**Total direct cost savings**	**0.1**	**0.2**	**0.3**	**0.5**
Indirect cost savings[2]				
Due to Mortality	<0.1	<0.1	<0.1	<0.1
Due to Morbidity	NA	NA	NA	NA
**Total indirect cost savings**	**<0.1**	**<0.1**	**<0.1**	**<0.1**
**Total cost savings**	**0.1**	**0.2**	**0.3**	**0.6**
**Cost savings following LDL-C and SBP reduction combined**				
Direct cost savings				
Hospital care	<0.1	<0.1	<0.1	0.2
Physician care	<0.1	<0.1	0.2	0.3
Drug	<0.1	0.2	0.4	0.7
**Total direct cost savings**	**0.2**	**0.4**	**0.6**	**1.2**
Indirect cost savings[2]				
Due to Mortality	<0.1	<0.1	<0.1	<0.1
Due to Morbidity	NA	NA	NA	NA
**Total indirect cost savings**	**<0.1**	**<0.1**	**<0.1**	**<0.1**
**Total cost savings**	**0.2**	**0.4**	**0.6**	**1.3**

[1] Data represent cardiovascular disease-related financial savings following reductions in LDL-cholesterol concentrations and systolic blood pressure with the consumption of 100 g/day pulses for men and women [3]. The very optimistic scenario is an estimate of potential savings when 50% of Canadian adults (≥18 years of age)

consume one 100 g/day serving of dietary pulses. The optimistic scenario is a medium-to-long-term pragmatic estimate of potential savings when 25% of adults in Canada consume pulse regularly. The pessimistic scenario is a practical short-to-medium-term estimate of cost savings that could follow the dietary pulse consumptions among 15% of adults. The very pessimistic scenario is an immediate estimate when 5% of Canadian adults adopt 100 g/day serving of pulses. LDL-C, LDL-cholesterol; NA, not available; SBP, systolic blood pressure. [2] Indirect costs only include values of lost production due to reduced working time associated with illness, injury, or premature death, and do not include any valuation of morbidity and mortality themselves.

Table S26. Potential annual savings in healthcare and related costs of cardiovascular disease among Canadian adults from 100 g/day dietary pulse intake in Quebec (Can $ million).[1]

	Scenario			
	**Very pessimistic**	**Pessimistic**	**Optimistic**	**Very optimistic**
**Cost savings following LDL-C reduction**				
Direct cost savings				
Hospital care	0.6	1.8	2.9	5.9
Physician care	0.8	2.5	4.2	8.4
Drug	2.5	7.6	12.7	25.4
**Total direct cost savings**	**4.0**	**11.9**	**19.8**	**39.7**
Indirect cost savings[2]				
Due to Mortality	<0.1	0.1	0.2	0.4
Due to Morbidity	NA	NA	NA	NA
**Total indirect cost savings**	**<0.1**	**0.1**	**0.2**	**0.4**
**Total cost savings**	**4.0**	**12.0**	**20.0**	**40.1**
**Cost savings following SBP reduction**				
Direct cost savings				
Hospital care	0.5	1.4	2.3	4.7
Physician care	0.7	2.0	3.3	6.7
Drug	2.0	6.0	10.1	20.2
**Total direct cost savings**	**3.2**	**9.5**	**15.8**	**31.5**
Indirect cost savings[2]				
Due to Mortality	<0.1	0.1	0.2	0.3
Due to Morbidity	NA	NA	NA	NA
**Total indirect cost savings**	**<0.1**	**0.1**	**0.2**	**0.3**
**Total cost savings**	**3.2**	**9.6**	**15.9**	**31.9**
**Cost savings following LDL-C and SBP reduction combined**				
Direct cost savings				
Hospital care	1.1	3.2	5.2	10.6
Physician care	1.5	4.5	7.5	15.1
Drug	4.5	13.6	22.8	45.6
**Total direct cost savings**	**7.2**	**21.4**	**35.6**	**71.2**
Indirect cost savings[2]				
Due to Mortality	<0.1	0.2	0.4	0.7
Due to Morbidity	NA	NA	NA	NA
**Total indirect cost savings**	**<0.1**	**0.2**	**0.4**	**0.7**
**Total cost savings**	**7.2**	**21.6**	**35.9**	**72.0**

[1] Data represent cardiovascular disease-related financial savings following reductions in LDL-cholesterol concentrations and systolic blood pressure with the consumption of 100 g/day pulses for men and women [3]. The very optimistic scenario is an estimate of potential savings when 50% of Canadian adults (≥18 years of age)

consume one 100 g/day serving of dietary pulses. The optimistic scenario is a medium-to-long-term pragmatic estimate of potential savings when 25% of adults in Canada consume pulse regularly. The pessimistic scenario is a practical short-to-medium-term estimate of cost savings that could follow the dietary pulse consumptions among 15% of adults. The very pessimistic scenario is an immediate estimate when 5% of Canadian adults adopt 100 g/day serving of pulses. LDL-C, LDL-cholesterol; NA, not available; SBP, systolic blood pressure. [2] Indirect costs only include values of lost production due to reduced working time associated with illness, injury, or premature death, and do not include any valuation of morbidity and mortality themselves.

Table S27. Potential annual savings in healthcare and related costs of cardiovascular disease among Canadian adults from 100 g/day dietary pulse intake in Saskatchewan (Can $ million).[1]

	Scenario			
	Very pessimistic	Pessimistic	Optimistic	Very optimistic
**Cost savings following LDL-C reduction**				
Direct cost savings				
Hospital care	0.1	0.3	0.4	0.9
Physician care	0.1	0.4	0.7	1.4
Drug	0.3	0.8	1.4	2.8
**Total direct cost savings**	**0.5**	**1.5**	**2.5**	**5.1**
Indirect cost savings[2]				
Due to Mortality	<0.1	<0.1	<0.1	0.1
Due to Morbidity	NA	NA	NA	NA
**Total indirect cost savings**	**<0.1**	**<0.1**	**<0.1**	**0.1**
**Total cost savings**	**0.5**	**1.5**	**2.6**	**5.1**
**Cost savings following SBP reduction**				
Direct cost savings				
Hospital care	0.1	0.2	0.4	0.7
Physician care	0.1	0.3	0.6	1.1
Drug	0.2	0.7	1.1	2.2
**Total direct cost savings**	**0.4**	**1.2**	**2.0**	**4.0**
Indirect cost savings[2]				
Due to Mortality	<0.1	<0.1	<0.1	<0.1
Due to Morbidity	NA	NA	NA	NA
**Total indirect cost savings**	**<0.1**	**<0.1**	**<0.1**	**<0.1**
**Total cost savings**	**0.4**	**1.2**	**2.0**	**4.1**
**Cost savings following LDL-C and SBP reduction combined**				
Direct cost savings				
Hospital care	0.2	0.5	0.8	1.6
Physician care	0.2	0.7	1.3	2.5
Drug	0.5	1.5	2.5	5.0
**Total direct cost savings**	**0.9**	**2.7**	**4.5**	**9.1**
Indirect cost savings[2]				
Due to Mortality	<0.1	<0.1	<0.1	<0.1
Due to Morbidity	NA	NA	NA	NA
**Total indirect cost savings**	**<0.1**	**<0.1**	**<0.1**	**<0.1**
**Total cost savings**	**0.9**	**2.7**	**4.6**	**9.2**

[1] Data represent cardiovascular disease-related financial savings following reductions in LDL-cholesterol concentrations and systolic blood pressure with the consumption of 100 g/day pulses for men and women [3]. The very optimistic scenario is an estimate of potential savings when 50% of Canadian adults (≥18 years of age)

consume one 100 g/day serving of dietary pulses. The optimistic scenario is a medium-to-long-term pragmatic estimate of potential savings when 25% of adults in Canada consume pulse regularly. The pessimistic scenario is a practical short-to-medium-term estimate of cost savings that could follow the dietary pulse consumptions among 15% of adults. The very pessimistic scenario is an immediate estimate when 5% of Canadian adults adopt 100 g/day serving of pulses. LDL-C, LDL-cholesterol; NA, not available; SBP, systolic blood pressure. [2] Indirect costs only include values of lost production due to reduced working time associated with illness, injury, or premature death, and do not include any valuation of morbidity and mortality themselves.

Table S28. Potential annual savings in healthcare and related costs of cardiovascular disease among Canadian adults from 100 g/day dietary pulse intake in Yukon (Can $ million).[1]

	Scenario			
	**Very pessimistic**	**Pessimistic**	**Optimistic**	**Very optimistic**
**Cost savings following LDL-C reduction**				
Direct cost savings				
Hospital care	<0.1	<0.1	<0.1	<0.1
Physician care	<0.1	<0.1	<0.1	<0.1
Drug	<0.1	<0.1	<0.1	<0.1
**Total direct cost savings**	**<0.1**	**<0.1**	**<0.1**	**0.1**
Indirect cost savings[2]				
Due to Mortality	<0.1	<0.1	<0.1	<0.1
Due to Morbidity	NA	NA	NA	NA
**Total indirect cost savings**	**<0.1**	**<0.1**	**<0.1**	**<0.1**
**Total cost savings**	**<0.1**	**<0.1**	**<0.1**	**0.1**
**Cost savings following SBP reduction**				
Direct cost savings				
Hospital care	<0.1	<0.1	<0.1	<0.1
Physician care	<0.1	<0.1	<0.1	<0.1
Drug	<0.1	<0.1	<0.1	<0.1
**Total direct cost savings**	**<0.1**	**<0.1**	**<0.1**	**0.1**
Indirect cost savings[2]				
Due to Mortality	<0.1	<0.1	<0.1	<0.1
Due to Morbidity	NA	NA	NA	NA
**Total indirect cost savings**	**<0.1**	**<0.1**	**<0.1**	**<0.1**
**Total cost savings**	**<0.1**	**<0.1**	**<0.1**	**0.1**
**Cost savings following LDL-C and SBP reduction combined**				
Direct cost savings				
Hospital care	<0.1	<0.1	<0.1	<0.1
Physician care	<0.1	<0.1	<0.1	<0.1
Drug	<0.1	<0.1	<0.1	<0.1
**Total direct cost savings**	**<0.1**	**<0.1**	**<0.1**	**0.2**
Indirect cost savings[2]				
Due to Mortality	<0.1	<0.1	<0.1	<0.1
Due to Morbidity	NA	NA	NA	NA
**Total indirect cost savings**	**<0.1**	**<0.1**	**<0.1**	**<0.1**
**Total cost savings**	**<0.1**	**<0.1**	**<0.1**	**0.2**

[1] Data represent cardiovascular disease-related financial savings following reductions in LDL-cholesterol concentrations and systolic blood pressure with the consumption of 100 g/day pulses for men and women [3]. The very optimistic scenario is an estimate of potential savings when 50% of Canadian adults (≥18 years of age)

consume one 100 g/day serving of dietary pulses. The optimistic scenario is a medium-to-long-term pragmatic estimate of potential savings when 25% of adults in Canada consume pulse regularly. The pessimistic scenario is a practical short-to-medium-term estimate of cost savings that could follow the dietary pulse consumptions among 15% of adults. The very pessimistic scenario is an immediate estimate when 5% of Canadian adults adopt 100 g/day serving of pulses. LDL-C, LDL-cholesterol; NA, not available; SBP, systolic blood pressure. [2] Indirect costs only include values of lost production due to reduced working time associated with illness, injury, or premature death, and do not include any valuation of morbidity and mortality themselves.

References

[1] Public Health Agency of Canada. EBIC Custom Report Generator. 2014. Available online: http://ebic-femc.phac-aspc.gc.ca/custom-personnalise/national.php?clear=1 (accessed on 02 December 2015).

[2] Statistics Canada. Health Care Consumer Price Index. Available online: http://www.statcan.gc.ca/tables-tableaux/sum-som/l01/cst01/econ161a-eng.htm (accessed on 14 June 2016).

[3] Health Canada. Eating Well with Canada's Food Guide (HC Pub.: 4651). Ottawa: Queen's Printer, 2007.
